# Revisiting metformin as a chemotherapy adjuvant: a systematic review and meta-analysis based on recent randomized-controlled trials

**DOI:** 10.3389/fonc.2026.1888506

**Published:** 2026-09-09

**Authors:** Bushra Wadi Bin Saddiq, Ahmed Salah Morad, Tanveer Nidal Khan, Joud Jamal Alshowaikhat, Jorie Nasser Alsharari, Renad Omar Bajri, Meral Jehad AlZayer, Saaid Mounzer Mouazen, Mohammed Abdulhameed Abutaleb, Suhaib Radi, Hassan Alwafi

**Affiliations:** 1 Faculty of Medicine and Surgery, Batterjee Medical College, Jeddah, Saudi Arabia; 2 College of Medicine, Alfaisal University, Riyadh, Saudi Arabia; 3 College of Medicine, King Saud Bin Abdulaziz University for Health Sciences, Jeddah, Saudi Arabia; 4 King Abdullah International Medical Research Center, Jeddah, Saudi Arabia; 5 Department of Internal Medicine, Division of Endocrinology, Ministry of the National Guard-Health Affairs, Jeddah, Saudi Arabia; 6 Department of Clinical Pharmacology and Toxicology, College of Medicine, Umm Al-Qura University, Makkah, Saudi Arabia

**Keywords:** adjuvant therapy, cancer, chemotherapy, drug repurposing, metformin, RCT, solid tumors, survival outcomes

## Abstract

**Objective:**

The primary objective is to assess metformin’s impact on progression free survival (PFS), overall survival (OS), and overall response rates (ORR). Secondary objectives included chemotherapy-related toxicity, glucose metabolism, and quality of life.

**Methods:**

A systematic review and meta-analysis were conducted following PRISMA guidelines and registered prospectively in PROSPERO. A comprehensive literature search was performed in November 2024 across PubMed, Google Scholar, Ovid MEDLINE, and ClinicalTrials.gov. Randomized controlled trials evaluating metformin as an adjunct to chemotherapy were included. The primary outcomes assessed were PFS, OS, and ORR; secondary outcomes included chemotherapy-related side effects, glucose metabolism, and quality of life. Data extraction and quality assessment were performed using standardized tools, and statistical analyses were conducted using RevMan 5.4 Heterogeneity was assessed using the *I²* statistic, and a random-effects model was applied when necessary.

**Results:**

Nineteen RCTs involving 3,502 participants were included. Subgroup analyses demonstrated a significant OS benefit when metformin (1500 mg/day) was combined with platinum-based chemotherapy (HR = 0.61, 95% CI 0.46–0.81; *p* = 0.0006). Metformin also significantly improved ORR in patients with breast cancer (OR = 1.86, 95% CI 1.19–2.92; *p* = 0.007) and in studies using a 500 mg/day dose (OR = 3.39, 95% CI 1.37–8.39; *p* = 0.008). Overall, metformin significantly improved ORR (OR = 1.59, 95% CI 1.02–2.46; *p* = 0.04). However, no significant improvements were observed in overall PFS (HR = 1.08, 95% CI 0.96–1.22; *p* = 0.19) or OS (HR = 1.08, 95% CI 0.90–1.30; *p* = 0.41). Grade ≥3 adverse events were comparable between groups (OR = 1.10, 95% CI 0.66–1.84; *p* = 0.72).

**Conclusion:**

Adjunctive metformin did not improve overall or PFS but was associated with a higher ORR, driven by increased complete responses, without increasing high-grade toxicity. A survival benefit was observed in exploratory subgroup analyses at a 1500 mg/day dose in platinum-based chemotherapy, warranting prospective trials to clarify these findings.

## Introduction

Cancer remains a major global health burden despite substantial progress in screening, diagnosis, and treatment. In 2022, an estimated 20 million new cancer cases and 9.7 million cancer-related deaths occurred worldwide, with solid malignancies accounting for most of this burden (1). Chemotherapy continues to be central to the management of many solid tumors, either alone or as part of multimodal treatment. However, its clinical benefit is frequently limited by dose-dependent toxicity, cumulative adverse effects, tumor heterogeneity, and the development of therapeutic resistance (2). These limitations have increased interest in drug repurposing strategies, especially for agents with known safety profiles, low cost, and biologically plausible anticancer effects. Metformin, a widely used biguanide and first-line pharmacologic therapy for type 2 diabetes mellitus, has therefore received significant attention as a potential adjunctive anticancer agent (3, 4). Its importance is particularly notable in solid tumors, for which metabolic reprogramming, altered insulin signaling, immune dysregulation, and treatment resistance play a major role in disease progression and recurrence (5–7).

The biological rationale for the use of metformin in oncology is supported by a number of interconnected mechanisms. At the cellular level, metformin can inhibit mitochondrial respiratory-chain complex I, leading to reduced oxidative phosphorylation, lower ATP availability, and activation of adenosine monophosphate-activated protein kinase (AMPK). AMPK activation may subsequently suppress mammalian target of rapamycin (mTOR) signaling, thereby limiting anabolic metabolism, protein synthesis, and tumor-cell proliferation (8). Beyond these direct effects, metformin may exert systemic and microenvironmental influences by reducing circulating insulin and glucose levels, attenuating inflammatory signaling, modulating antitumor immune responses, and affecting metabolic interactions between tumor cells and cancer-associated fibroblasts (5, 6, 8). Experimental data further indicate that metformin can increase sensitivity to antineoplastic treatments as well as disrupt adaptive mechanisms associated with chemotherapy resistance (2, 8). However, such properties seem to be context-dependent and might differ depending on the type of cancer, metabolic profile, drug exposure, and host glycemic status.

Clinical evidence evaluating metformin as an anticancer adjunct remains inconclusive. Early observational studies and subsequent meta-analyses reported associations between metformin use and reduced cancer incidence or improved survival, particularly among patients with diabetes (9–14). However, observational evidence is inherently limited by confounding factors, immortal-time bias, variations in diabetes severity, and inconsistent concurrent regimens. To resolve these uncertainties, several systematic reviews and meta-analyses synthesizing clinical trial data have emerged (15–17). While these secondary syntheses have enriched the literature, their findings remain mixed; some suggest localized benefits in specific cancer subtypes, whereas others demonstrate no OS or PFS benefit (15–17). Furthermore, existing meta-analyses are often limited by combining heterogeneous non-randomized designs, focusing exclusively on single organ sites, or failing to isolate metformin’s specific additive effect within standardized, chemotherapy-based regimens. Consequently, the clinical value of adding metformin to chemotherapy in patients with solid tumors remains uncertain. To address these gaps and map the clinical trial evidence landscape, this systematic review and meta-analysis synthesizes data exclusively from randomized controlled trials (RCTs) to evaluate the efficacy, safety, and clinical outcomes of adjuvant metformin combined with chemotherapy in solid malignancies.

## Methods

### Review of the literature

This systematic review followed the Preferred Reporting Items for Systematic Reviews and Meta-Analyses (PRISMA) model guidelines so as to ensure transparency (18). The study protocol was registered prospectively with the International Prospective Register of Systematic Reviews (PROSPERO), *a priori* with the following ID: CRD42024592273 (19). Owing to the nature of the study, ethical approval was not required. In November 2024, a comprehensive systematic search was conducted using the following databases: (1) PubMed, (2) Google Scholar, (3) Ovid MEDLINE, and (4) ClinicalTrials.gov. No limitations to language, geographical location or publication date were applied. To optimize search results, a combination of Medical Subject Heading (MeSH) terms, including Metformin (MeSH ID: D008687), Antineoplastic Agents (MeSH ID: D000971), and Neoplasms (MeSH ID: D009369), free text fields and Boolean operators were used. The search strategy included the following keywords: (metformin) AND (chemotherapy) AND (cancer OR neoplasm OR tumor) AND (overall survival OR progression-free survival OR response rate OR adverse events OR quality of life) AND (randomized controlled trial OR RCT). Based on the PICOS (Population, Intervention, Comparison, Outcome, Setting) criteria, studies were further considered for review and analysis (20).

### Methodology for selecting studies

For inclusion into our systematic review, the studies had to meet the following criteria: (1) study design should be RCTs, (2) cancer patients of any age without any restrictions on the type or grade of the cancer, (3) study participants treated with standard chemotherapy as the primary intervention; with use of metformin as an adjunct to chemotherapy as a comparator in the same study, (4) studies meeting the outcomes as stated: (a) Primary outcome: improvement in cancer-related outcomes such as tumor shrinkage, PFS, OS, and ORR.; (b) Secondary outcomes: impact on glucose metabolism, reduction in chemotherapy-induced side effects, quality of life, and any adverse effects related to metformin use. Studies were excluded from our systematic review based on the following criteria: (1) other study designs including non-randomized trials, case reports, reviews, or conference papers, (2) studies not using standard chemotherapy and metformin as an adjunct to the chemotherapy as the interventional modalities, (3) studies without a comparator group or studies comparing patients to healthy individuals, (4) studies that did not report on any of the predefined outcomes of interest.

### Process of screening and data extraction

All study articles were obtained following database searching and amassed into a Google spreadsheet. Four investigators (JJ, JN, BB, AM) independently screened for titles and abstracts using the web-tool “Rayyan— search web and mobile application for systematic review” (21). In case of any discrepancies, the article then proceeded to a full-text review. Subsequently, the full-texts of the included studies were screened against the set eligibility criteria by 2 investigators (RB, SM). Any disagreement was resolved by consensus or by consulting a senior reviewer.

Thereafter, data extraction was performed independently by three investigators (TK, MJ, MA) and manually recorded into a separate spreadsheet. This standardized spreadsheet included variables such as study characteristics, participant demographics, intervention details, and outcome measures. In cases where data were inconsistent or unclear, “GetData Graph Digitizer” was used to get data directly from the respective graphs or images (22). When data regarding standard deviations were unaccounted for, they were calculated using the standard error of subgroups or confidence intervals.

To ensure accuracy and to avoid duplication, the data retrieved was double-checked and underwent a secondary review by a separate set of investigators (BB, AM). All extracted variables were compiled into a final datasheet for further analysis.

Potential population overlap between the included studies by Skinner et al. (23) (NRG-LU001, NCT02186847) and Tsakiridis et al. (24) (ALMERA, NCT02115464) was explicitly evaluated. While both trials evaluated similar clinical interventions, they were conducted independently by distinct multi-center cooperative groups (NRG Oncology in the United States and the Ontario Clinical Oncology Group in Canada, respectively) utilizing separate patient cohorts. We confirmed that no participant populations overlapped or were double-counted across these studies in any pooled analyses.

### Assessment of quality and bias risk

The quality and bias risk of each of the included studies were assessed independently by 2 reviewers (TK, AM) using the Cochrane Risk of Bias 2 (RoB 2) tool. with the following five domains evaluated: bias arising from the randomization process, bias due to deviations from intended interventions, bias due to missing outcome data, bias in the measurement of the outcome, and bias in the selection of the reported result. Each domain was assessed as ‘low risk,’ ‘some concerns,’ or ‘high risk’ according to RoB 2 guidelines, and an overall risk of bias judgment was assigned for each included study (25).

### Meta-analysis of the included data

This Meta-analysis was conducted to utilize findings from the studies that were included in the search to determine the effects of metformin as an adjunct to chemotherapy in cancer patients. Statistical analysis was conducted using the Revman Manager (RevMan) Version 5.4 software, which aided in the pooling of the study results as well as the evaluation of overall treatment effects (26). Heterogeneity across studies was examined using the Cochrane-based Q statistic and *I²* test, which determined the type of model to be further used in the analysis. Due to anticipated clinical and methodological heterogeneity across the included studies (e.g., differences in patient populations, cancer types, and treatment regimens), a random-effects model was used for all pooled analyses regardless of statistical heterogeneity.

PFS, and OS, and ORR were the primary outcomes assessed in this meta-analysis. The secondary outcomes were the impact of metformin on glucose metabolism, its role in mitigating chemotherapy-related side effects, and its overall effect on quality of life. Standardized mean differences (SMD) for continuous outcomes and odds ratio (OR) for categorical variables were used to express the effects of treatment; each had a 95% confidence interval (CI). P-values below 0.05 were regarded as statistically significant.

Subgroup analysis was implemented on feasible studies to explore variations in outcomes based on cancer type, chemotherapy regimen, and study design. Sensitivity analysis was also conducted in general to discern if exclusion of any of the trials led to a significant change in the results. Additionally, due to the availability in an adequate number of studies, a funnel plot could be charted so as to assess the publication bias.

## Results

### Search results

**Figure 1** illustrates the PRISMA flow chart that depicts the steps of study inclusion in our review. Using our search strategy, we retrieved a total of 178 studies from four different databases and registers. Twenty duplicates were found and removed. Further screening based on title and abstract excluded 140 studies. The full text of the remaining 45 studies was sought for retrieval, and only six could not be accessed. After assessing the eligibility of the studies through full text, 20 studies were excluded due to various reasons. Finally, 19 studies were included in our systematic review and meta-analysis (23, 24, 27–43).

**Figure 1:**
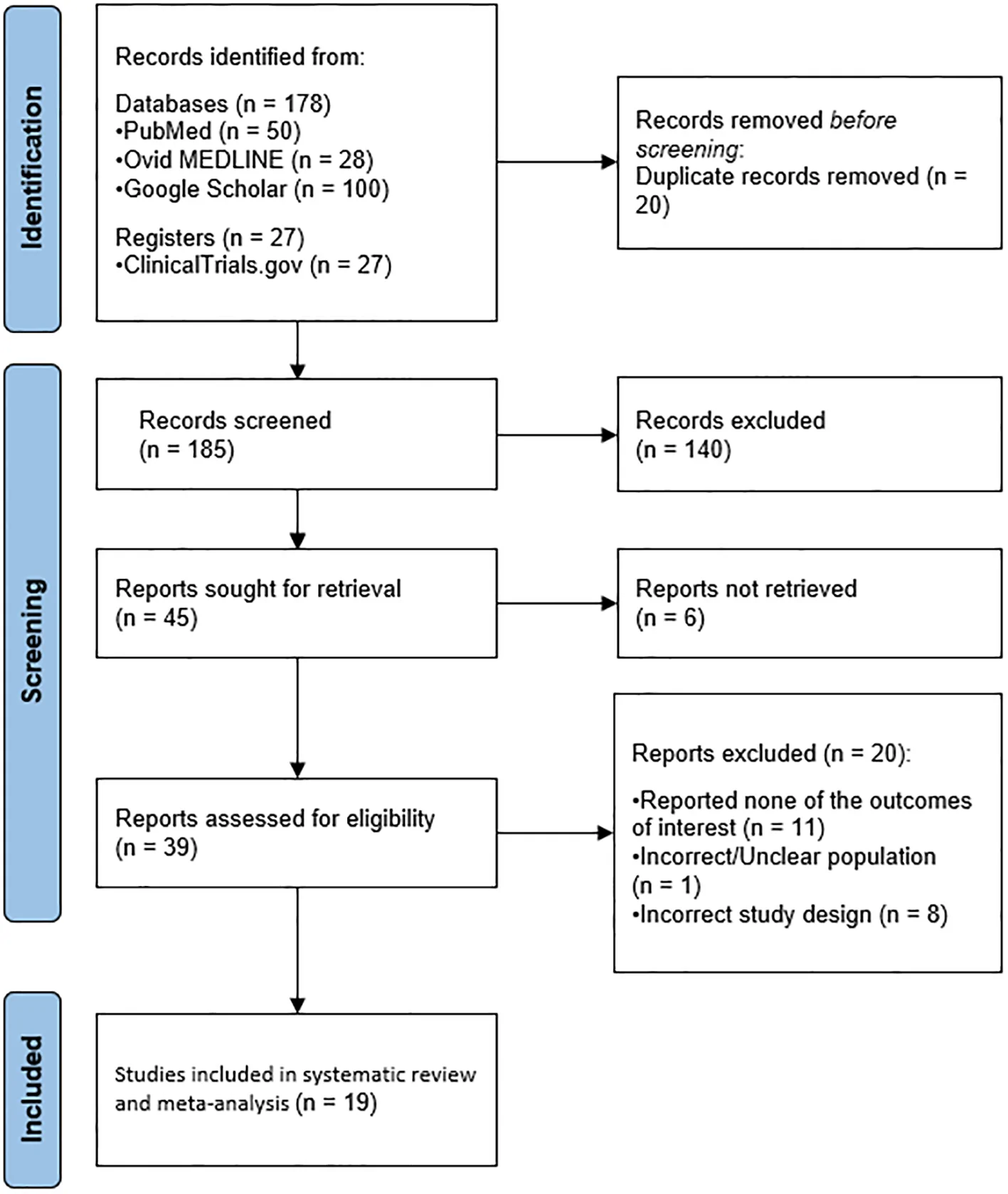
PRISMA 2020 flow diagram illustrating the study selection process. Flowchart demonstrating the identification,screening,eligibility assessment,and final inclusion of randomized controlled trials (RCTs) evaluating metformin as an adjuvant therapy in cancer treatment. PRISMA, Preferred Reporting Items for Systematic Reviews and Meta-Analyses; n, number of studies/records; RCT, randomized controlled trial.

### Summary of study characteristics

A total of 19 RCTs (23, 24, 27–43) were analyzed, involving 3502 participants with a mean age in the intervention group of 57.9 ± 11.6 years and 57.2 ± 11.8 years in the control group. The gender distribution across the studies was 39.6% male and 60.4% female in the intervention group, whereas 46.3% male and 53.7% female in the control group, as shown in **Table 1**. Notably, the phase-III trial by Singh et al. (2016) contributed a substantial proportion of the total sample size (n = 1,958; 55.9% of the total cohort), thereby heavily driving the pooled demographic distribution and statistical weighting of the control group.

**Table 1:** Study characteristics.

Study ID	Country	Blinding	Design	Follow-up	Number of participants (metformin/Control)	Type of metformin	Mean age of participants (± SD)		Gender (*n* (%); M/F)		Cancer type	Mean daily dose/maintenance dose of metformin	Frequency of dosage	Comparator group regimen
							Metformin	Control	Metformin	Control				
Skinner 2021 (23)	US, Canada, Israel	Open-label	Parallel-group RCT	27.7 months	167 (86/81)	N/A	N/A	N/A	M: 49 (57.0%) F: 37 (43.0%)	M: 48 (59.3%) F: 33 (40.7%)	Lung Cancer	2000mg	Daily	Chemotherapy + Platinum
Martin-Castillo 2018 (27)	Spain	Open-label	Parallel-group Phase II RCT	N/A	58 (29/29)	N/A	45.3 ± 9.8	48.5 ± 11.8	M: 0 (0%) F: 29 (100%)	M: 0 (0%) F: 29 (100%)	Breast Cancer	850mg	Twice Daily	Chemotherapy
Pimentel 2019 (28)	Canada	Double blind	Phase II RCT	2.25 months	40 (22/18)	N/A	N/A	N/A	M: 0 (0%) F: 22 (100%)	M: 0 (0%) F: 18 (100%)	Breast Cancer	850mg	Twice Daily	Chemotherapy
Wilmink 2015 (29)	Netherlands	Double blind	Parallel-group Phase II RCT	28.1 months	121 (60/61)	N/A	64 ± 8	65 ± 9	M: 34 (57.0%) F: 26(43.0%)	M: 27 (44.0%) F: 34(56.0%)	Pancreatic Cancer	Week 1: 1000mg Followed by: 2000mg	Daily	Chemotherapy
Marrone 2018 (30)	United States	Open-label	Single-center, parallel-group RCT	6.9 months	24 (18/6)	N/A	N/A	N/A	M: 7 (38.9%) F: 11 (61.1%)	M: 2 (33.3%) F: 4 (66.7%)	Lung Cancer	1000mg	Twice Daily	Chemotherapy + Platinum
Yoon 2023 (31)	Republic of Korea	Double-blind	Phase II RCT	4 Months	81 (38/43)	Standard metformin	55.9 ± 10.6	55.5 ± 11.8	M: 22 (57.9%%) F: 16 (42.1%)	M: 21 (48.8%) F: 22 (51.2%)	Glioblastoma multiforme (GBM)	Week 1: 1000 mg Week 2: 1500 mg Week 3: 2000 mg	Daily	Chemotherapy
Essa 2022 (32)	Egypt and Saudi Arabia	N/A	RCT	6 months	107 (57/50)	Standard metformin	49.56 ± 12.53	48.40 ± 12.61	M: 0 (0%) F: 57 (100%)	M: 0 (0%) F: 50 (100%)	Breast Cancer	500mg	Twice Daily	Chemotherapy
Rabea 2021 (33)	Bahrain	Double-blind	Parallel-group RCT	6 months	50 (25/25)	Immediate-release formulation.	48.8 ± 9.4	49.2 ± 11.7	M: 0 (0%) F: 25 (100%)	M: 0 (0%) F: 25 (100%)	Breast Cancer	1000 mg	Twice Daily	Chemotherapy
Vernieri 2019 (34)	Italy	Open-label	Phase III multicenter RCT	60.4 months	120 (76/44)	Standard metformin	67.2 ± 5.3	67.9 ± 5.2	M: 50 (65.8%%) F: 26 (34.2%)	M: 23 (52.3%) F: 21 (47.7%)	Colon Cancer	N/A	N/A	Chemotherapy
Reni 2016 (35)	Italy	Open-label	Parallel-group.	10.4 months	60 (31/29)	Immediate-release formulation.	64.0 ± 8.4	63.0 ± 7.9	M: 17 (55%) F: 14 (45%)	M: 13 (45%) F: 16 (55%)	Pancreatic Cancer	2000 mg	Daily	Chemotherapy
Sayed 2015 (36)	Egypt	Open-label	Prospective RCT	12 months	30 (15/15)	Immediate-release formulation	56.0 ± 8.1	52.0 ± 7.9	M: 12 (80%) F: 3 (20%)	M: 13 (86.7%) F: 2 (13.3%)	Lung Cancer	500mg	Daily	Chemotherapy
Tsakiridis 2021 (24)	Canada	N/A	Phase-II RCT	N/A	54 (26/28)	Immediate-release metformin	65.9 ± 8.1	65.3 ± 7.3	M: 12 (46.2%) F: 14 (53.8%)	M: 12 (42.9%) F: 16 (57.1%)	N/A	1000 mg	Twice Daily	Chemotherapy
Hegazy 2024 (37)	Egypt	Open-label	Phase-II RCT	15.9 months	100 (50/50)	Immediate-release formulation.	51.18 ± 12.64	51.59 ± 15.45	M: 18 (36%) F: 32 (64%)	M: 30 (60%) F: 20 (40%)	Lymphoma	1000 mg	Daily	Chemotherapy
Nanni 2018 (38)	N/A	Open-label	Phase-II multicenter RCT	12 months	126 (59/67)	Standard metformin	N/A	N/A	M: 0 (0%) F: 59 (100%)	M: 0 (0%) F: 67 (100%)	Breast cancer	1000 mg	Twice Daily	Chemotherapy
Zheng 2019 (39)	China	Open-label	RCT	30 months	44 (20/24)	Standard metformin	53.55 ± 9.20	52.88 ± 8.77	M: 0 (0%) F: 20 (100%)	M: 0 (0%) F: 24 (100%)	Ovarian Cancer	850 mg	Daily	Chemotherapy + Platinum
Lee 2020 (40)	South Korea	open-label	Phase II RCT	32.4 months	164 (81/83)	Standard metformin	N/A	N/A	M: 72 (88.9%) F: 9 (11.1%)	M: 67 (80.7%) F: 16 (19.3%)	Lung Cancer	Week 1: 500 mg Followed by: 1000 mg	Twice Daily	Platinum
Singh 2016 (41)	United States	N/A	Phase-III randomized trial	78 months	1958 (155/1691)	Standard metformin	61 ± 8.5	57.5 ± 11.4	M: 66 (42.6%) F: 49 (57.4%)	M: 876 (51.8%) F: 815 (48.2%)	Colon Cancer	N/A	N/A	Chemotherapy
El-Khayat 2021 (42)	Egypt	N/A	Prospective randomized study	N/A	118 (59/59)	Standard metformin	N/A	N/A	M: 0 (0%) F: 59 (100%)	M: 0 (0%) F: 59 (100%)	Breast Cancer	850mg	Week 1: Daily Followed by: Twice Daily	Chemotherapy
Barakat 2022 (43)	Egypt	Open-label	Single-center RCT	N/A	80 (40/40)	Standard metformin	49.14 ± 11.22	47.13 ± 10.53	M: 0 (0%) F: 40 (100%)	M: 0 (0%) F: 40 (100%)	Breast Cancer	Week 1: 500 mg Week 2: 500 mg Followed by: 1000 mg	Week 1: Twice Daily Week 2: Three times daily Followed by: 1000 mg twice daily	Chemotherapy
					Total: 3502		Total: 57.9 ± 11.6	Total: 57.2 ± 11.8	Total: M: 359 (39.6%) F: 548 (60.4%)	Total: M: 1132 (46.3%) F: 1311 (53.7%)				

Values are presented as mean ± standard deviation (SD) for continuous variables and absolute counts with percentages (n [%]) for categorical variables. Total participant counts and pooled baseline metrics across all 19 studies are presented in the summary row. Data designated as “N/A” indicate variables or outcomes that were not reported in the original published studies. Study ID, First author and publication year; RCT, randomized controlled trial; M, Male; F, Female; SD, standard deviation; N/A, Not available; mg, milligrams.

The trials were conducted across multiple countries, with five studies from Egypt (32, 36, 37, 42, 43), three from the United States (23, 30, 41), two from Italy (34, 35), and others spanning other regions including Canada (23, 24, 28), the Netherlands (29), South Korea (31, 40), Israel (23), Bahrain (33), China (39) and Saudi Arabia (32). Among these, 2 studies were multinational in scope (23, 32).

In terms of methodological structure, six trials followed a parallel design (23, 27, 29, 30, 33, 35), while four were prospective randomized studies (32, 36, 39, 42). Among these, two studies were single-centers (30, 43), and two were multicenter trials (34, 38). Follow-up duration ranged from 2.25 months to a maximum of 102 months (8.5 years). Notably, four studies did not specify a follow-up period (24, 27, 42, 43).

Various cancer types and stages were examined in the studies, with breast cancer being the most studied malignancy, followed by lung, pancreas, colon, bladder, cervical, ovarian, and lymphoma. The metformin doses varied across papers, with some trials employing fixed doses, while others followed a titration dose approach. Most studies used standard metformin formulations (31, 32, 34, 38–43), while a few tested immediate-release formulations (24, 33, 35–37).

Primary outcomes assessed were mainly PFS, OS, and ORR with varying methodologies across studies. Comparator groups included chemotherapy alone, chemotherapy plus platinum-based agents, and in some cases, placebo agents. The daily metformin dose ranged from 250 mg to 2000 mg, administered in various dosage frequencies.

### Quality assessment

Randomized controlled trials were assessed independently using the Cochrane Risk of Bias 2 (RoB 2) tool (25) with the following five domains evaluated: bias arising from the randomization process, bias due to deviations from intended interventions, bias due to missing outcome data, bias in the measurement of the outcome, and bias in the selection of the reported result. Each domain was assessed as ‘low risk,’ ‘some concerns,’ or ‘high risk’ according to RoB 2 guidelines, and an overall risk of bias judgment was assigned for each included study.

Overall, 9 of the 19 included randomized controlled trials (47.4%) were judged to be at low risk of bias, 7 studies (36.8%) raised some concerns, and 3 studies (15.8%) were considered to be at high risk of bias. Most studies demonstrated a low risk of bias in the domains of missing outcome data and outcome measurement, while concerns were most frequently identified in the randomization process, deviations from intended interventions, and selection of the reported result.

However, three studies warranted additional consideration. Martin-Castillo et al. (2018) was judged to have a high risk of bias primarily due to missing outcome data, which may have influenced the estimated treatment effect. Vernieri et al. (2019) demonstrates a high risk of bias due to concerns regarding the randomization process, stemming from the insufficient reporting of sequence generation and allocation concealment. Similarly, Singh et al. (2016) was judged to be at high risk because of limitations in describing the randomization process, besides additional concerns regarding deviations from intended interventions, raising concerns of selection bias. Despite the limitations, the overall assessment suggests that the majority of included studies maintained a fair to high quality evidence standard with minimal risk of systematic bias (**Figures 2**, **3**). Domain-level and overall risk of bias judgments for each individual trial are detailed in **Supplementary Table 1**. Furthermore, detailed trial design parameters, randomization methods, and allocation concealment strategies for all 19 included RCTs are summarized in **Supplementary Table 2**.

**Figure 2:**
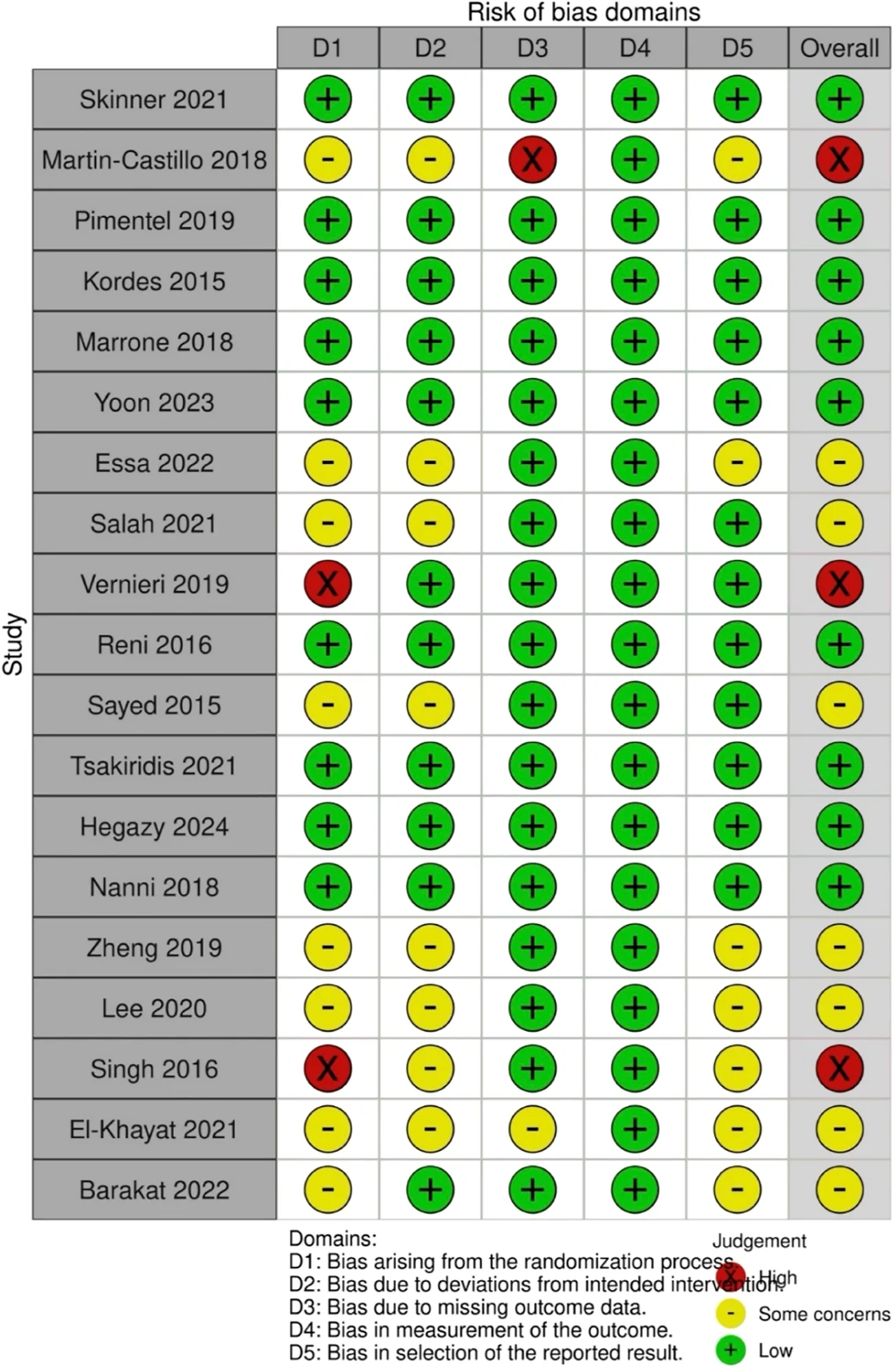
Traffic-light plot of the Cochrane RoB 2 assessment. Individual domain-level judgments and overall risk of bias evaluations assessed using the Cochrane Risk of Bias 2 tool across all 19 included trials. Domains: D1: Bias arising from the randomization process; D2: Bias due to deviations from intended interventions; D3: Bias due to missing outcome data; D4: Bias in measurement of the outcome; D5: Bias in selection of the reported result. Judgments: Green circular icon with plus sign (+) = Low risk of bias; Yellow circular icon with dash (-) = Some concerns; Red circular icon with “X” (X) = High risk of bias. RoB 2, Cochrane Risk of Bias 2 tool; RCT, randomized controlled trial.

**Figure 3:**
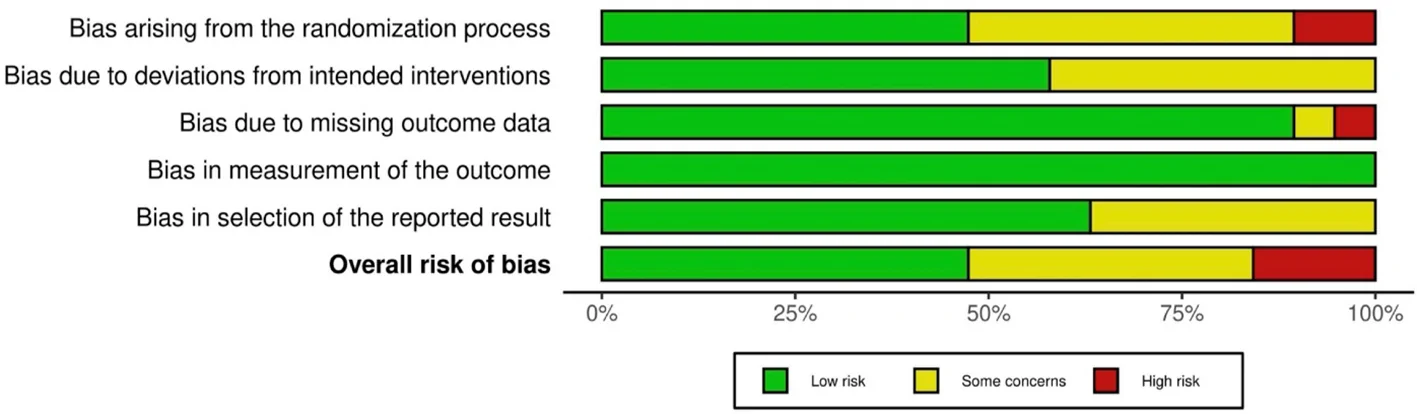
Summary graph of the Cochrane RoB 2 assessment. Bar chart displaying the overall percentage distribution of risk of bias judgments across the five individual domain assessments and overall risk using the Cochrane Risk of Bias 2 (RoB 2) tool for all 19 included trials. Color Coding: Green bar segment = Low risk of bias; Yellow bar segment = Some concerns; Red bar segment = High risk of bias. RoB 2, Cochrane Risk of Bias 2 tool; RCT, randomized controlled trial.

### Outcomes

**Table 2** summarizes the outcomes of the meta-analysis whether related to efficacy or safety. The main outcomes evaluated for efficacy were the PFS, the OS, and the ORR in patients. The presence of ≥ grade 3 adverse events was the outcome used to assess the safety.

**Table 2:** Summary of outcomes.

Outcomes		Number of studies	Number of patients (*n*)		Heterogeneity		Model used (Fixed/Random)	Effect size			
			Metformin	Control	*p*	*I²* (%)		HR	OR	95% CI	*p*
Efficacy	PFS (23, 24, 28, 29, 31, 32, 35, 36, 38–40)	11	521	522	0.55	0	Random	1.08	-	[0.96, 1.22]	0.19
	OS (23, 24, 29–31, 34–36, 38, 41)	10	574	2075	0.21	25	Random	1.08	-	[0.90, 1.30]	0.41
	ORR (27–32, 35–38, 40, 42, 43)	14	576	590	0.07	38	Random	-	1.59	[1.02, 2.46]	0.04*
	Complete response (CR) (27, 30, 31, 37, 42, 43)	6	234	227	0.55	0	Random	-	2.03	[1.12, 3.66]	0.02*
	Partial response (PR) (27–29, 31, 35, 36, 43)	7	245	245	0.19	31	Random	-	1.14	[0.62, 2.08]	0.67
Safety	Grade ≥ 3 adverse events	8	437	1963	0.07	45	Random	-	1.10	[0.66, 1.84]	0.72

*P-value is significant (< 0.05).

Heterogeneity Assessment (I^2^): I^2^ = 0 - 25% indicates low heterogeneity; I^2^ = 26 – 50% indicates moderate heterogeneity; I^2^ > 50% indicates substantial heterogeneity.

Effect Measure Selection: Hazard ratios (HR) express effect estimates for time-to-event outcomes (PFS and OS), whereas odds ratios (OR) express effect estimates for dichotomous/binary outcomes (ORR, CR, PR, and ≥ Grade 3 adverse events).

### Progression-free survival

**Figure 4** demonstrates the results of the meta-analysis of disease progression-free survival. Hazard ratio (HR) was used to measure the effect size. The analysis revealed no significant difference between metformin and standard treatment in terms of PFS (HR = 1.08, *p* = 0.19). Notable variability was observed in Salah 2021 (HR = 0.31, 95% CI: [0.06, 1.54]) and Tsakiridis 2021 (HR = 2.42, 95% CI: [1.00, 5.88]). However, due to small sample sizes (n = 50 and n = 54, respectively) and resulting high standard errors, both studies received low statistical weights (0.6% and 1.8%) and did not significantly distort the overall pooled effect.

**Figure 4:**
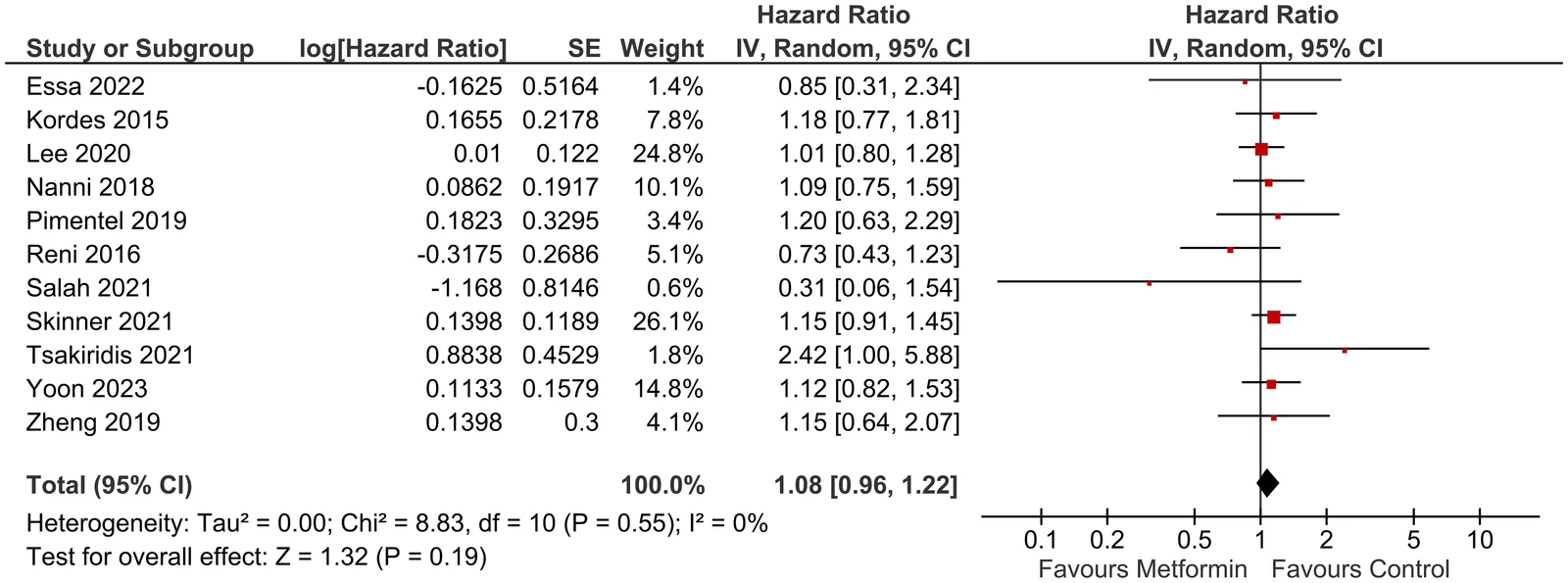
Forest plot of hazard ratios for PFS comparing metformin with control. Pooled effect estimates calculated using a random-effects model (Inverse Variance method). Red squares indicate individual study estimates with size proportional to study weight; horizontal lines represent 95% CIs. The black diamond shows the overall pooled effect. HR, hazard ratio; PFS, progression-free survival; SE, standard error; CI, confidence interval; IV, inverse variance.

### Overall survival

**Figure 5** shows the results of the meta-analysis of OS. Hazard ratio was used to measure the effect size. The analysis revealed no significant difference between metformin and standard treatment in terms of OS (HR = 1.08, *p* = 0.41). Although overall statistical heterogeneity was low-to-moderate (I^2^ = 25%), variations in individual study estimates and confidence intervals likely reflect clinical heterogeneity across trials, including differences in cancer types, metformin dosing, and concurrent chemotherapy regimens.

**Figure 5:**
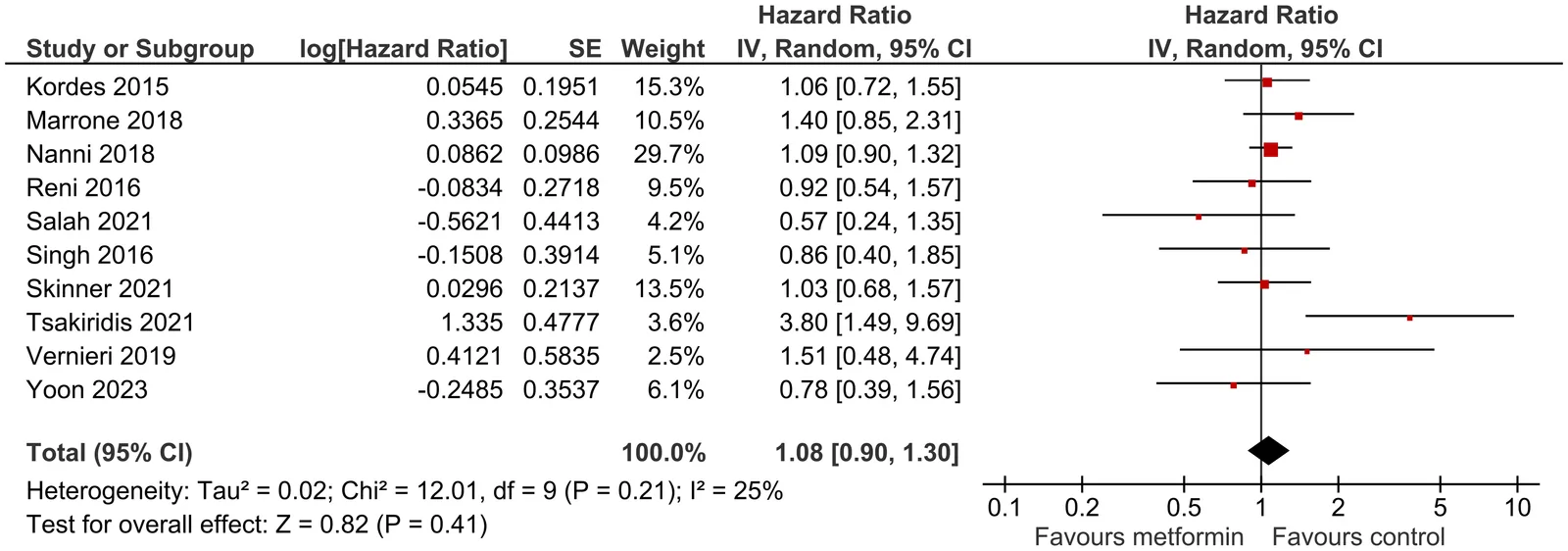
Forest plot of hazard ratios for OS comparing metformin vs. control. Pooled effect estimates calculated using a random-effects model (Inverse Variance method). Red squares indicate individual study estimates with size proportional to study weight; horizontal lines represent 95% CIs. The black diamond shows the overall pooled effect. HR, hazard ratio; OS, overall survival; SE, standard error; CI, confidence interval; IV, inverse variance.

### Response rates

**Figure 6** shows the results of the meta-analysis of CR, PR, and ORR. OR was used to measure the effect size. The analysis revealed that the overall and CR were significant higher in the metformin group when compared to the control group (OR = 1.59, *p* = 0.04, OR = 2.03, *p* = 0.02; respectively). However, no significant difference was found in the PRR between metformin and control groups (OR = 1.14, *p* = 0.67).

**Figure 6:**
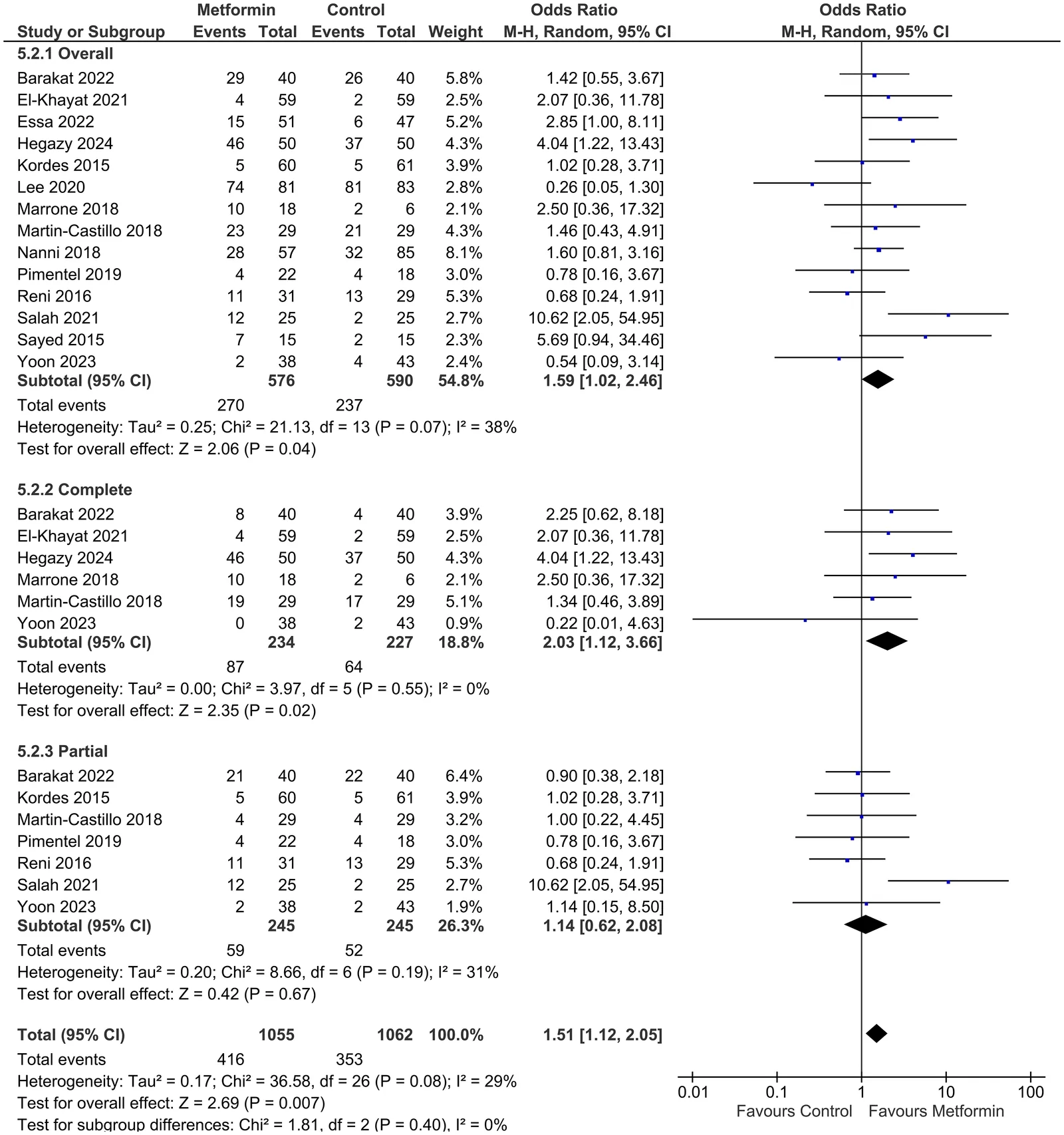
Forest plot of odds ratios for ORR, CR, and PR comparing metformin vs. control. Subgroup meta-analysis assessing ORR,complete response (CR),and partial response (PR) using a random-effects model (Mantel-Haenszel method). Blue squares represent individual study estimates with horizontal lines showing 95% CIs. Black diamonds represent pooled subgroup and overall odds ratios. OR, odds ratio; ORR, overall response rate; CR, complete response; PR, partial response; M-H, Mantel-Haenszel method; CI, confidence interval.

### Subgroup analysis

Subgroup analysis was conducted in the following aspects. Firstly, subgrouping the studies based on the different standard treatments used in the studies was performed. For PFS, Subgroup analysis demonstrated a statistically significant outcome in the chemotherapy group (**Supplementary Figure 1**, HR = 1.23, 95% CI: 1.02–1.49, *p* = 0.03), whereas no significant effect was observed in the chemotherapy + platinum subgroup (**Supplementary Figure 1**, HR = 1.13, 95% CI: 0.90–1.42, *p* = 0.28) or the platinum subgroup (**Supplementary Figure 1**, HR = 1.01, 95% CI: 0.80–1.28, *p* = 0.93). No statistically significant subgroup differences were observed (**Supplementary Figure 1**, Chi² = 1.60, 95% CI [1.01, 1.29], *p* = 0.45, *I²* = 0%). For OS, there was no statistically significant effect in the chemotherapy group (HR = 1.07, 95% CI: 0.81–1.43, *p* = 0.63) or the chemotherapy + platinum subgroup (HR = 1.17, 95% CI: 0.85–1.61, *p* = 0.34). However, a significant benefit was noted in the platinum subgroup (HR = 0.61, 95% CI: 0.46–0.81, *p* = 0.0006), with significant subgroup heterogeneity (**Figure 7**, Chi² = 11.30, 95% CI [0.78, 1.29], *p* = 0.004, *I²* = 82.3%).

**Figure 7:**
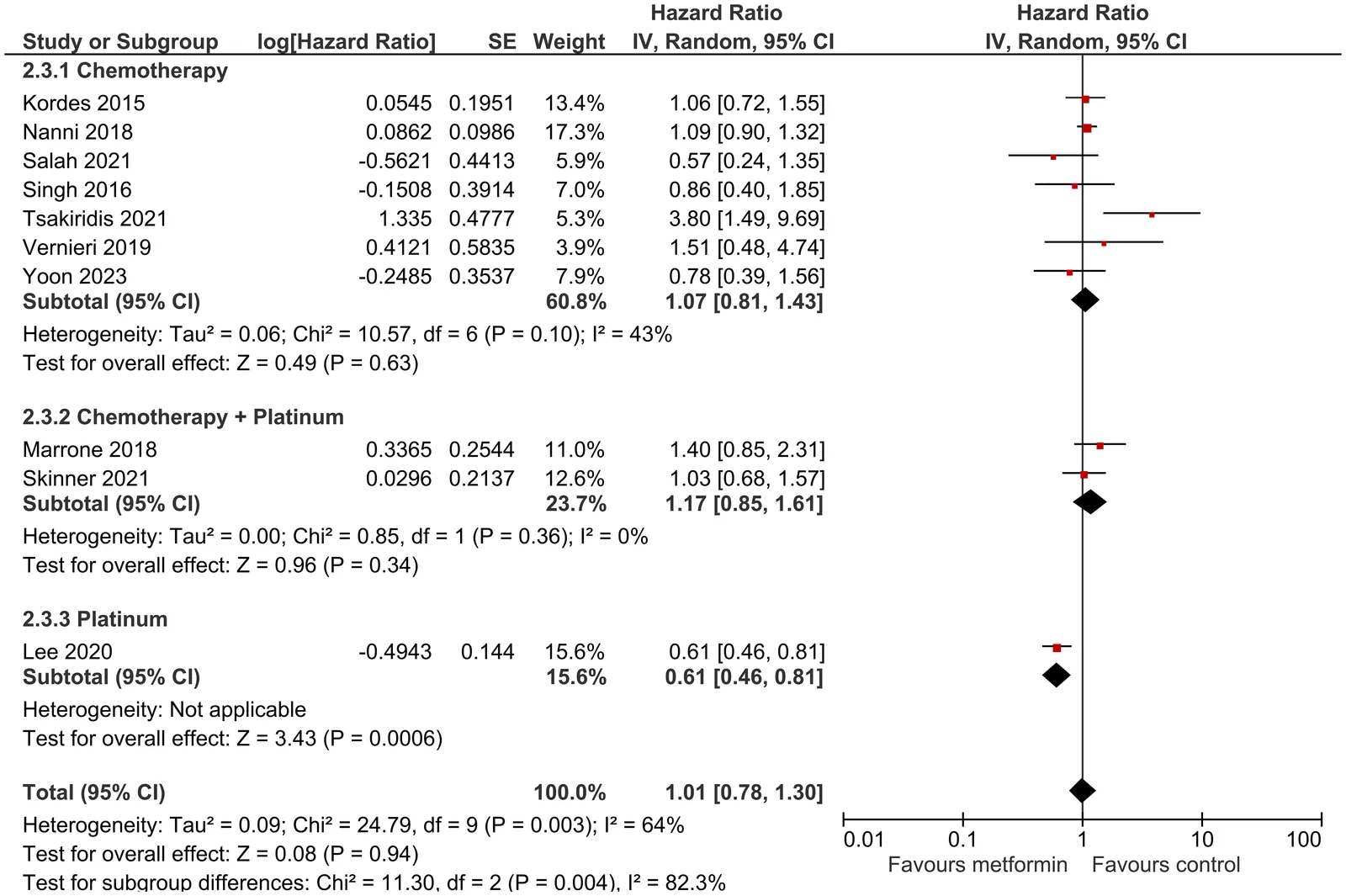
Subgroup analysis of hazard ratios for OS by standard chemotherapy regimen. Subgroup meta-analysis comparing metformin vs. control across different concurrent chemotherapy regimens (chemotherapy alone,chemotherapy + platinum,and platinum alone) using a random-effects model (Inverse Variance method). Red squares represent individual study estimates with horizontal lines showing 95% CIs. Black diamonds represent pooled subgroup and overall hazard ratios. HR, hazard ratio; OS, overall survival; SE, standard error; IV, inverse variance; CI, confidence interval.

The second subgroup analysis was according to the mean daily dose of metformin. For PFS, there was no statistically significant effect in the 500 mg (**Supplementary Figure 2**, HR = 0.85, 95% CI: 0.31–2.34, *p* = 0.75), 850 mg (**Supplementary Figure 2**, HR = 1.20, 95% CI: 0.63–2.29, *p* = 0.58), or 1000 mg subgroups (**Supplementary Figure 2**, HR = 1.06, 95% CI: 0.87–1.28, *p* = 0.58). However, a significant difference was observed in the 2000 mg subgroup (**Supplementary Figure 2**, HR = 1.19, 95% CI: 1.01–1.40, *p* = 0.04), with no significant subgroup heterogeneity (**Supplementary Figure 2**, Chi² = 1.14, *p* = 0.77, *I²* = 0%). For OS, there was no statistically significant difference in OS for the 1000 mg (HR = 1.28, 95% CI: 0.78–2.08, *p* = 0.33) or 2000 mg subgroups (HR = 1.00, 95% CI: 0.77–1.30, *p* = 0.99). However, a significant reduction in HR was observed in the 1500 mg subgroup (**Figure 8**, HR = 0.61, 95% CI: 0.46–0.81, *p* = 0.0006), with significant subgroup heterogeneity (Chi^2^ = 9.48, *p* = 0.009, *I^2^* = 78.9%). Furthermore, there was a statistically significant higher ORR in the 500 mg subgroup (**Figure 9**, OR = 3.39, 95% CI: 1.37–8.39, *p* = 0.008). However, no statistically significant differences were observed in the 850 mg (OR = 1.32, 95% CI: 0.57–3.05, *p* = 0.52), 1000 mg (OR = 1.93, 95% CI: 0.89–4.21, *p* = 0.10), or 2000 mg subgroups (OR = 0.74, 95% CI: 0.36–1.55, *p* = 0.43.

**Figure 8:**
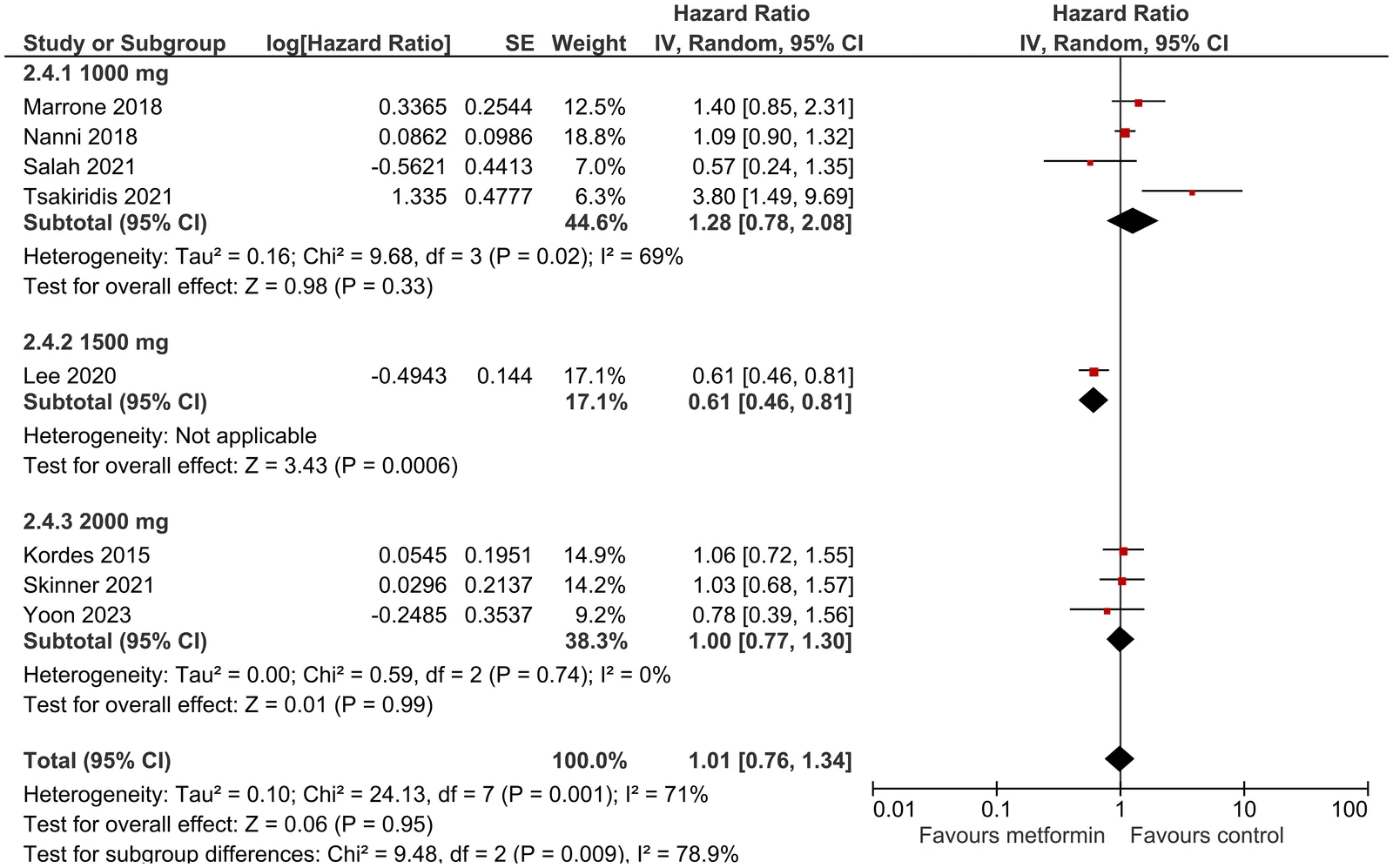
Subgroup analysis of hazard ratios for OS stratified by mean daily metformin dose. Subgroup meta-analysis comparing metformin vs. control across different daily dose tiers (1000 mg,1500 mg,and 2000 mg) using a random-effects model (Inverse Variance method). Red squares represent individual study estimates with horizontal lines showing 95% CIs. Black diamonds represent pooled subgroup and overall hazard ratios. HR, hazard ratio; OS, overall survival; mg, milligrams; SE, standard error; IV, inverse variance; CI, confidence interval.

**Figure 9:**
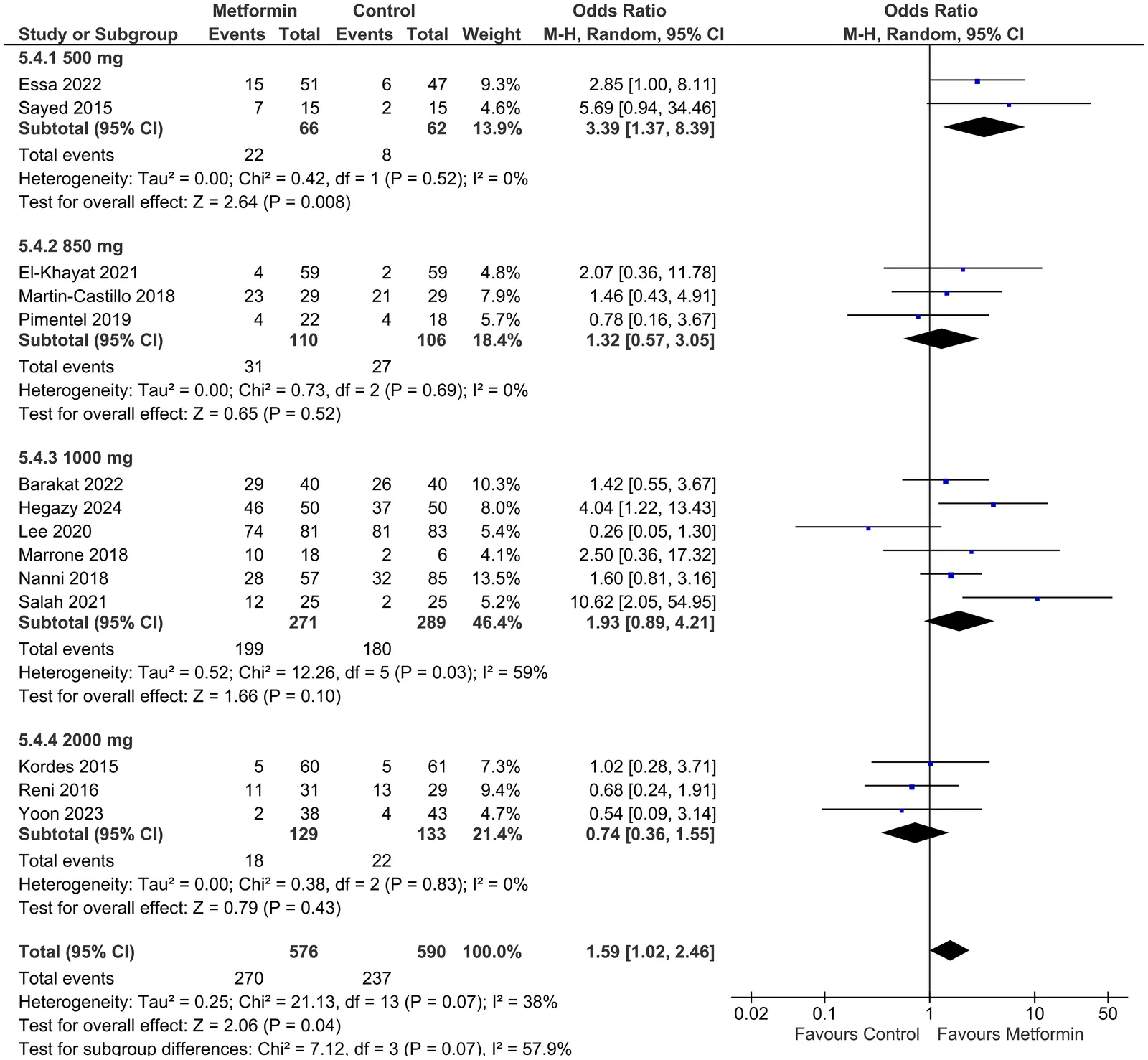
Subgroup analysis of OR for ORR stratified by mean daily metformin dose. Subgroup meta-analysis assessing overall response rate comparing metformin vs. control across daily dosage tiers (500 mg,850 mg,1000 mg,and 2000 mg) using a random-effects model (Mantel-Haenszel method). Blue squares represent individual study estimates with horizontal lines showing 95% CIs. Black diamonds represent pooled subgroup and overall odds ratios. OR, odds ratio; ORR, overall response rate; mg, milligrams; M-H, Mantel-Haenszel method; CI, confidence interval.

Subgrouping based on cancer type showed no statistically significant difference in ORR for lung cancer (OR = 1.38, 95% CI: 0.32–6.03, *p* = 0.67) or pancreatic cancer (OR = 0.79, 95% CI: 0.35–1.78, *p* = 0.58). However, a statistically significant increase in ORR was observed in the breast cancer subgroup (**Figure 10**, OR = 1.86, 95% CI: 1.19–2.92, *p* = 0.007). However, no statistically significant differences in OS across any of the evaluated tumor types: lung cancer (**Supplementary Figure 3**, HR = 1.17, 95% CI: 0.85–1.61, *p* = 0.34), breast cancer (**Supplementary Figure 3**, HR = 1.06, 95% CI: 0.88–1.28, *p* = 0.56), pancreatic cancer (**Supplementary Figure 3**, HR = 1.01, 95% CI: 0.74–1.37, *p* = 0.96), and colon cancer (**Supplementary Figure 3**, HR = 1.02, 95% CI: 0.54–1.94, *p* = 0.94). No significant subgroup heterogeneity was observed (**Supplementary Figure 3**, Chi² = 0.47, *p* = 0.93, *I²* = 0%).

**Figure 10:**
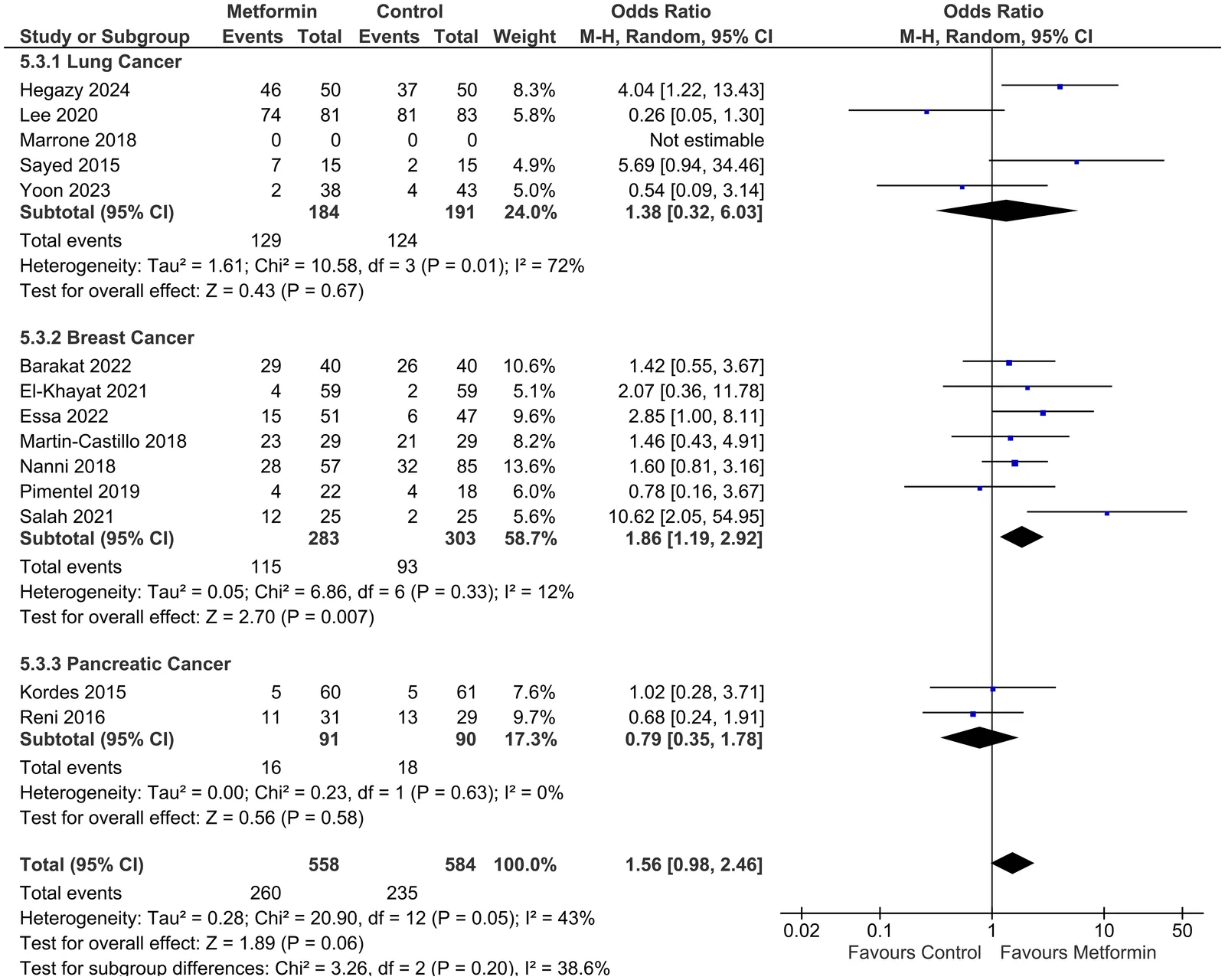
Subgroup analysis of odds ratios for ORR stratified by cancer type. Subgroup meta-analysis assessing overall response rate comparing metformin vs. control across distinct malignancy types (lung cancer,breast cancer,and pancreatic cancer) using a random-effects model (Mantel-Haenszel method). Blue squares represent individual study estimates with horizontal lines showing 95% CIs. Black diamonds represent pooled subgroup and overall odds ratios. OR, odds ratio; ORR, overall response rate; M-H, Mantel-Haenszel method; CI, confidence interval.

Lastly, subgroup analysis based on metformin formulation demonstrated no statistically significant differences in ORR for either the Standard formulation subgroup (**Supplementary Figure 4**, OR = 1.34, 95% CI: 0.75–2.39, *p* = 0.32) or the Immediate Release (IR) subgroup (**Supplementary Figure 4**, OR = 3.22, 95% CI: 0.89–11.66, *p* = 0.08), with low subgroup heterogeneity (**Supplementary Figure 4**, Chi² = 1.48, P = 0.22, *I²* = 32.3%).

### Publication bias

Funnel plots of the studies for PFS, OS, and ORR showed a symmetrical distribution suggesting no significant publication bias to be present in the studies (**Supplementary Figure 5**).

### Adverse events

When safety was taken into consideration, there was no significant difference in the incidence of ≥ Grade 3 adverse events between the metformin group and the control group (**Figure 11**, OR = 1.10, *p* = 0.72).

**Figure 11:**
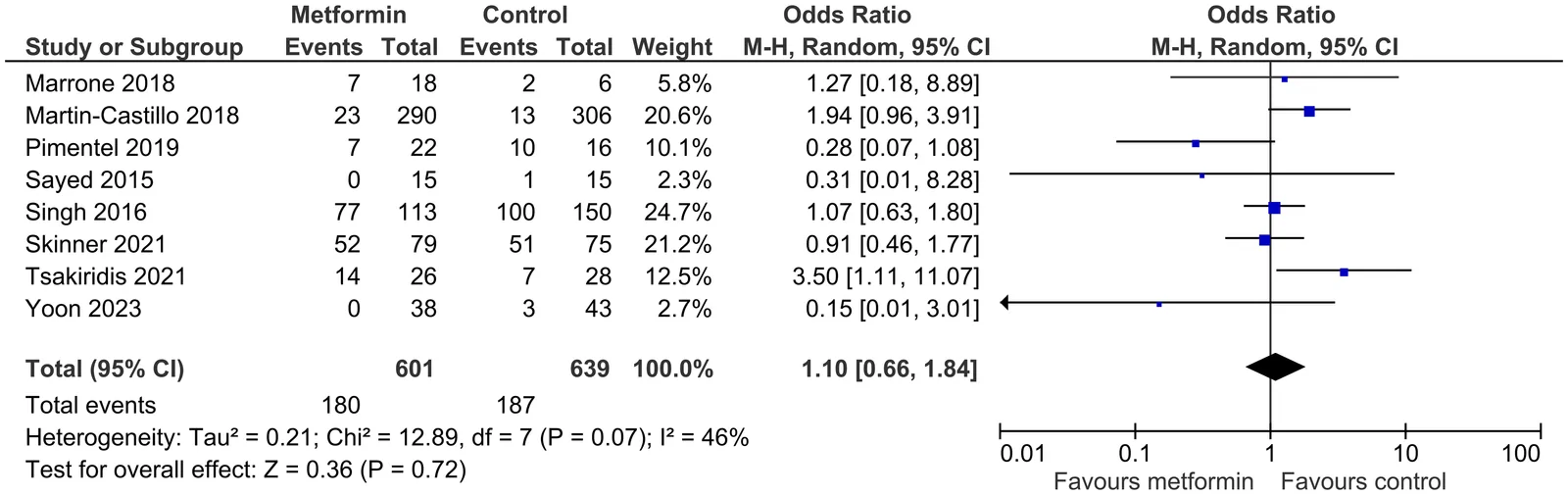
Forest plot of OR for adverse events comparing metformin vs. control. Meta-analysis evaluating the overall incidence of adverse events comparing metformin vs. control using a random-effects model (Mantel-Haenszel method). Blue squares represent individual study estimates with horizontal lines showing 95% CIs; size is proportional to study weight. The black diamond represents the pooled overall odds ratio. OR, odds ratio; M-H, Mantel-Haenszel method; CI, confidence interval.

## Discussion

This systematic review and meta-analysis reassessed the efficacy of metformin as an addition to standard cancer therapy across several malignancies, with a focus on PFS, OS, response outcomes, and safety. Although no overall improvement in survival outcomes was observed, metformin was linked to better treatment response results. These findings are broadly consistent with previous meta-analyses, which found limited evidence of a survival benefit associated with metformin therapy in cancer settings (16).

The observed improvement in treatment response outcomes may provide support for metformin’s predicted anticancer mechanisms, which have been examined in prior investigations. Metformin may influence tumor growth and response to therapy via activation of the AMPK pathway and suppression of mTOR signaling (8). However, despite these predicted mechanisms and the reported improvement in response outcomes, no corresponding improvement in survival outcomes was found in the overall analysis. The increase in objective response rate (ORR) was not accompanied by improvements in PFS or OS. As a result, while the reported response benefits may imply improved short-term tumor response, they do not necessarily translate into long-term clinical benefit, especially considering the heterogeneity of cancer types, treatment settings, and therapeutic regimens included in this analysis.

Subgroup analyses offered additional information about possible differences between clinical settings. Subgroup analyses revealed potential differences based on chemotherapy regimen; however, the trials that used a 1500 mg/day metformin dose were the same as those using platinum-based chemotherapy, resulting in identical effect estimates (HR = 0.61, p = 0.0006). Because these subgroup analyses were based on the same underlying trials, the observed survival benefit cannot be attributed independently to metformin dose or chemotherapy regimen. As a result, these findings should be viewed as exploratory and taken with caution, rather than proof of a dose- or regimen-specific effect. Furthermore, the chemotherapy subgroup had significantly lower PFS in the metformin arm (HR = 1.23, p = 0.03), suggesting an increased risk of disease progression. Given the absence of a similar finding in the overall analysis and the various subgroup comparisons conducted, this isolated result should be viewed with caution, as it could be a chance finding rather than an adverse effect. Similarly, differences were seen between metformin dose and cancer type, with breast cancer studies showing better response outcomes (28–32). However, these subgroup findings should be carefully evaluated because changes in study characteristics may have influenced the observed effects.

Several studies have also suggested that metformin may have direct anti-cancer effects by suppressing cellular proliferation, angiogenesis, and metastatic potential, as well as modulating the tumor microenvironment (44). These proposed mechanisms may help to explain the positive response results found in some of the studies included in this analysis. However, the therapeutic relevance of these effects is unknown due to the lack of a consistent survival benefit in the total pooled analysis.

The safety findings in this review were reassuring. The addition of metformin to conventional treatment did not increase the number of severe adverse events. This conclusion is consistent with earlier studies reported in this review and supports metformin’s favorable tolerability profile in cancer settings (16).

Several restrictions should be addressed when interpreting these results. The studies included a variety of cancer types, disease situations, chemotherapeutic regimens, metformin dosages, formulations, and follow-up times. Although subgroup analyses were conducted depending on chemotherapy regimen, metformin dose, cancer type, and formulation, clinical heterogeneity may have influenced the pooled values. As a result, the overall findings should be interpreted with caution, while the subgroup findings should be considered exploratory. Furthermore, certain subgroup analyses were based on a small number of studies, which may have influenced the reliability of the findings. Variability between studies could also be explained through differences in trial methods and study design. Furthermore, metformin dose was not randomized between studies, and differences in cancer types and treatment regimens between dose subgroups could have resulted in trial-level confounding. As a result, any apparent dose-related differences should not be interpreted as indicating a true dose-response relationship. Additionally, our literature search relied on a selected set of databases, and the exclusion of standard clinical trial repositories such as EMBASE and Cochrane CENTRAL must be acknowledged as a limitation. While we implemented a broad search strategy across PubMed, Google Scholar, and Ovid, this database selection may introduce a potential for database-selection bias and limit the identification of unindexed literature, which could influence publication bias assessments. As a result, some eligible randomized controlled trials may not have been identified, and the pooled estimates should be evaluated in light of this limitation.

Overall, the existing evidence does not show that metformin treatment provides a consistent survival benefit across all cancer populations studied. Although metformin improved objective response rates, these improvements were not associated with better PFS or OS. Nonetheless, the improved response outcomes reported in the overall study and in selected subgroups indicate that additional well-designed randomized controlled trials are required to better define the clinical settings in which metformin may be most effective.

## Conclusion

In this comprehensive meta-analysis, adjunctive metformin did not significantly improve OS or PFS but enhanced the ORR-driven by increased complete responses-without raising high-grade (≥ Grade 3) toxicity. Low heterogeneity across endpoints and a consistently low risk of bias supports these findings. Exploratory subgroup analyses suggested potential differences according to cancer type and dose, particularly at lower metformin doses (500–1000 mg/day), in breast cancer patients, and at 1500 mg/day alongside platinum-based chemotherapy. However, given the lack of OS benefits and the inherent clinical heterogeneity across the included literature, these subgroup observations should be interpreted with caution. While not universally recommended, metformin’s ability to boost tumor response in select settings warrants well-powered, subtype-focused randomized trials with standardized regimens and optimized dosing to confirm these tentative findings.

## Data Availability

The original contributions presented in the study are included in the article/**Supplementary Material**. Further inquiries can be directed to the corresponding author.
