# Correction: Revisiting metformin as a chemotherapy adjuvant: a systematic review and meta-analysis based on recent randomized-controlled trials

**DOI:** 10.3389/fonc.2026.2000444

**Published:** 2026-09-22

**Authors:** Bushra Wadi Bin Saddiq, Ahmed Salah Morad, Tanveer Nidal Khan, Joud Jamal Alshowaikhat, Jorie Nasser Alsharari, Renad Omar Bajri, Meral Jehad AlZayer, Saaid Mounzer Mouazen, Mohammed Abdulhameed Abutaleb, Suhaib Radi, Hassan Alwafi

**Affiliations:** 1 Faculty of Medicine and Surgery, Batterjee Medical College, Jeddah, Saudi Arabia; 2 College of Medicine, Alfaisal University, Riyadh, Saudi Arabia; 3 College of Medicine, King Saud Bin Abdulaziz University for Health Sciences, Jeddah, Saudi Arabia; 4 King Abdullah International Medical Research Center, Jeddah, Saudi Arabia; 5 Department of Internal Medicine, Division of Endocrinology, Ministry of the National Guard-Health Affairs, Jeddah, Saudi Arabia; 6 Department of Clinical Pharmacology and Toxicology, College of Medicine, Umm Al-Qura University, Makkah, Saudi Arabia

**Keywords:** adjuvant therapy, cancer, chemotherapy, drug repurposing, metformin, RCT, solid tumors, survival outcomes

Affiliation “Faculty of Medicine and Surgery, Batterjee Medical College, Jeddah, Saudi Arabia” was erroneously included in the paper. This affiliation has now been removed for author Hassan Alwafi.

The original version of this article has been updated.

