# Supplementary material for: Revisiting metformin as a chemotherapy adjuvant: a systematic review and meta-analysis based on recent randomized-controlled trials
Source: Front Oncol. 2026 Sep 9;16:1888506. doi: 10.3389/fonc.2026.1888506 (PMC13639577; doi:10.3389/fonc.2026.1888506)
Supplement: Supplementary file 1 [file DataSheet1.pdf]

Supplementary figures:

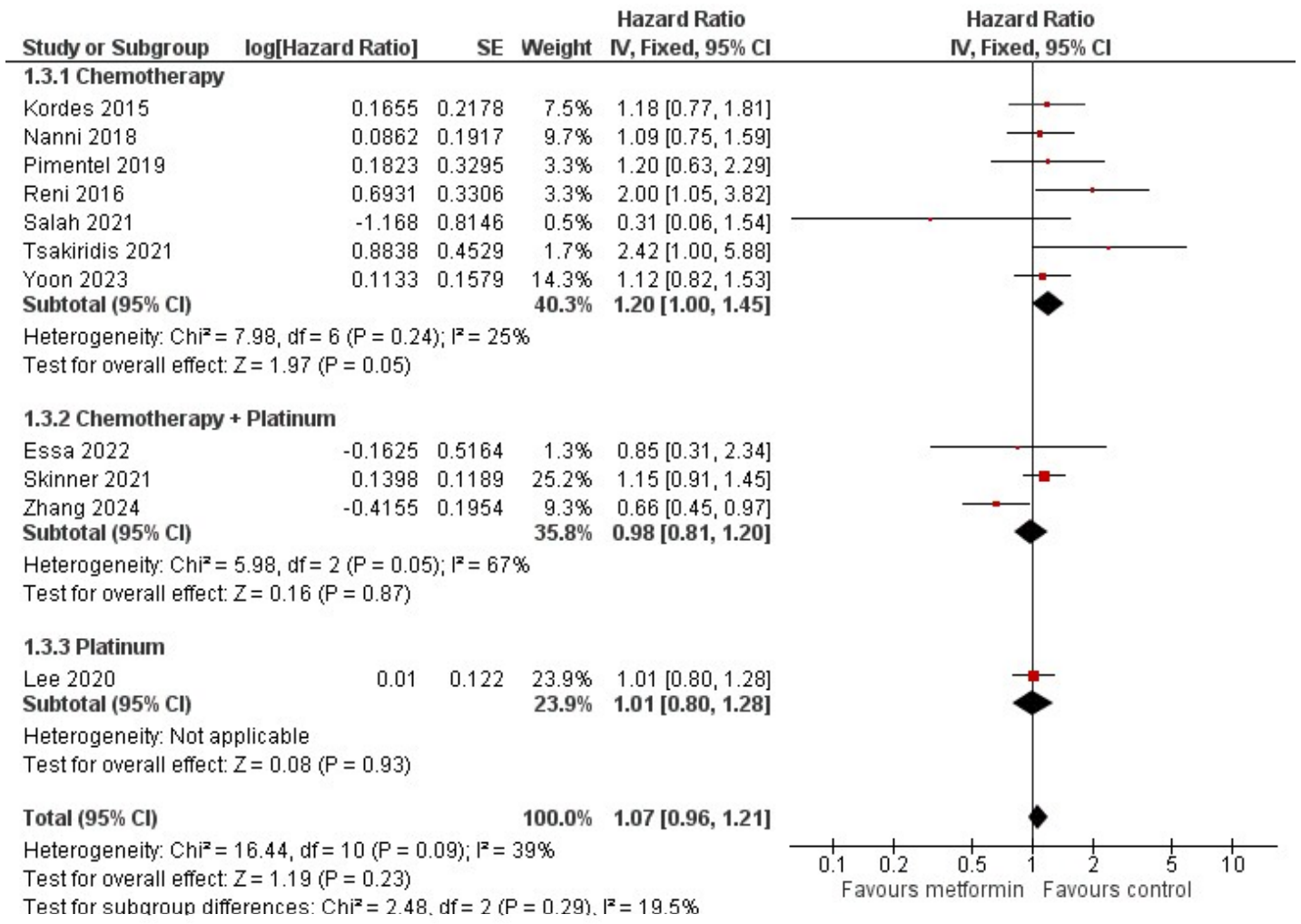

Figure S1. HR of PFS of metformin vs control based on standard chemotherapy used

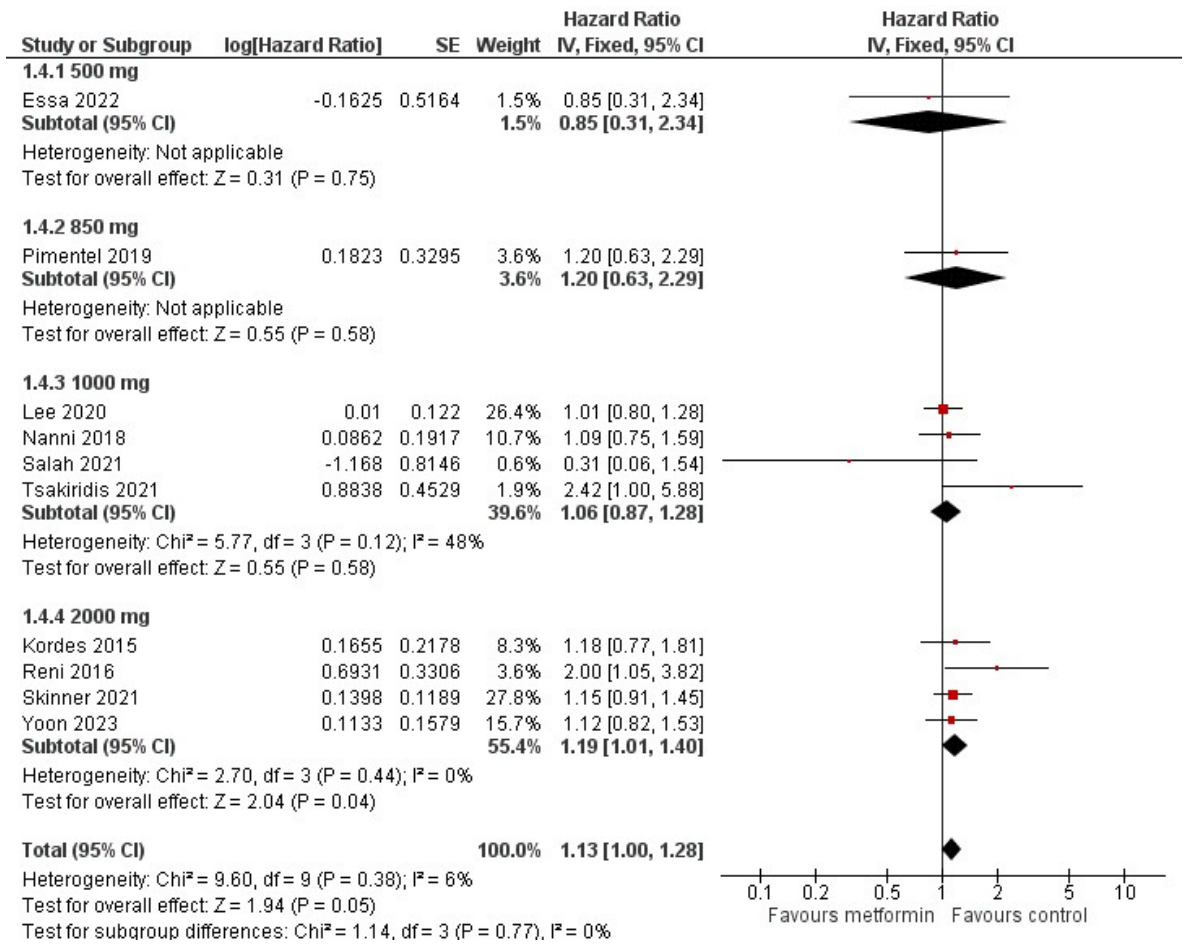

Figure S2. HR of PFS of metformin vs control based on mean daily dose

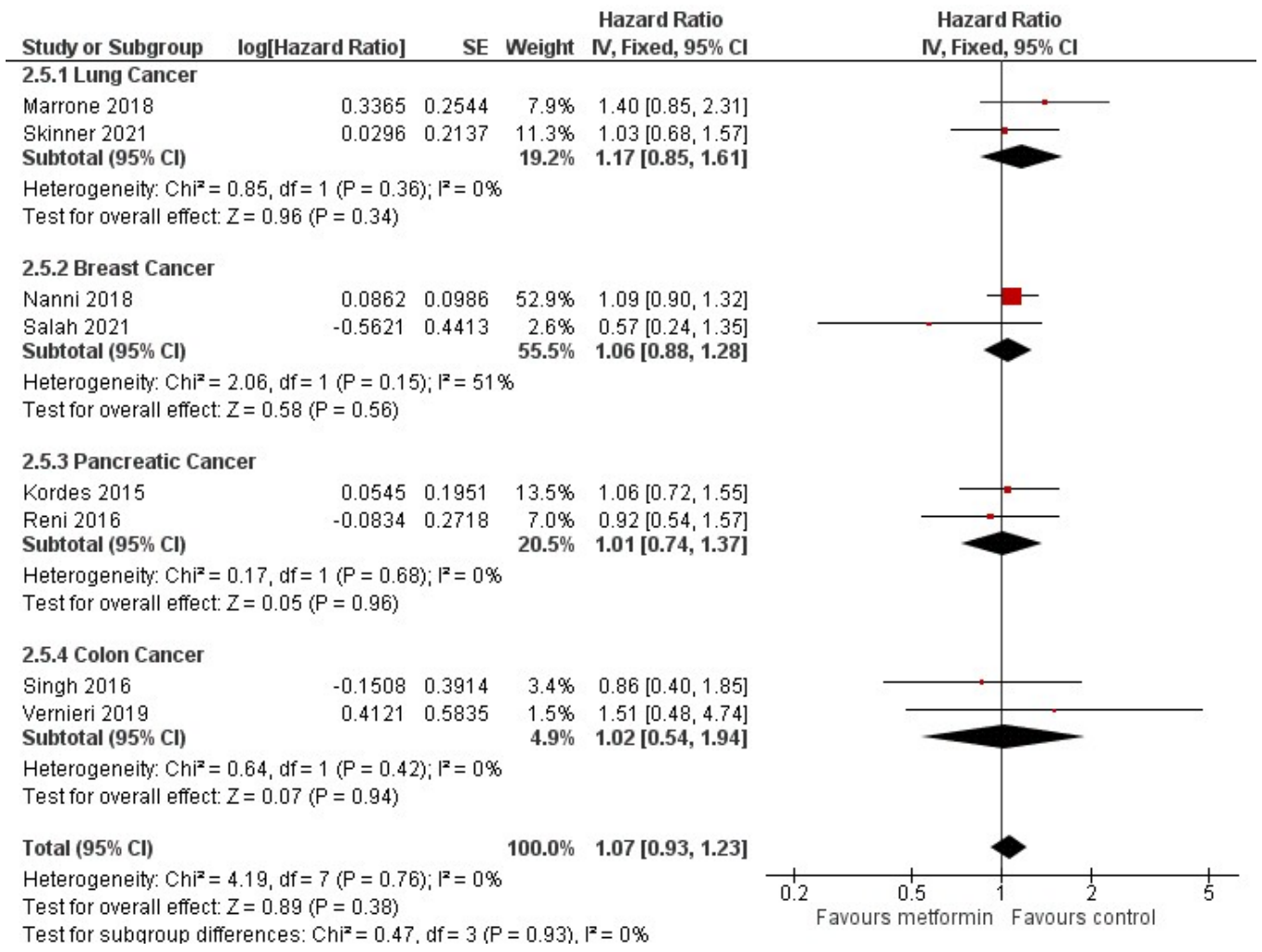

Figure S3. HR of OS of metformin vs control based on cancer type

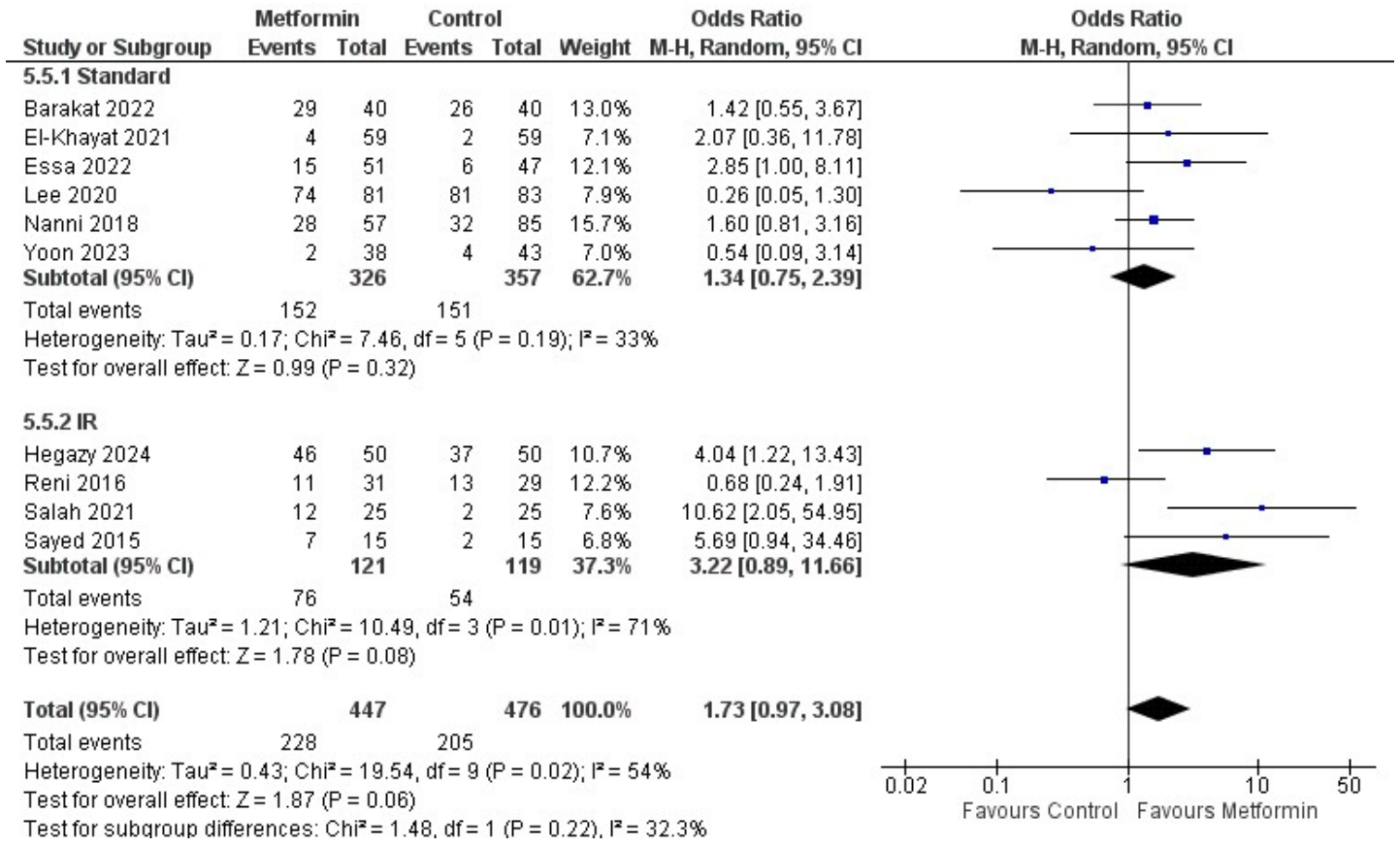

Figure S4. HR of OS of metformin vs control based on metformin formulation

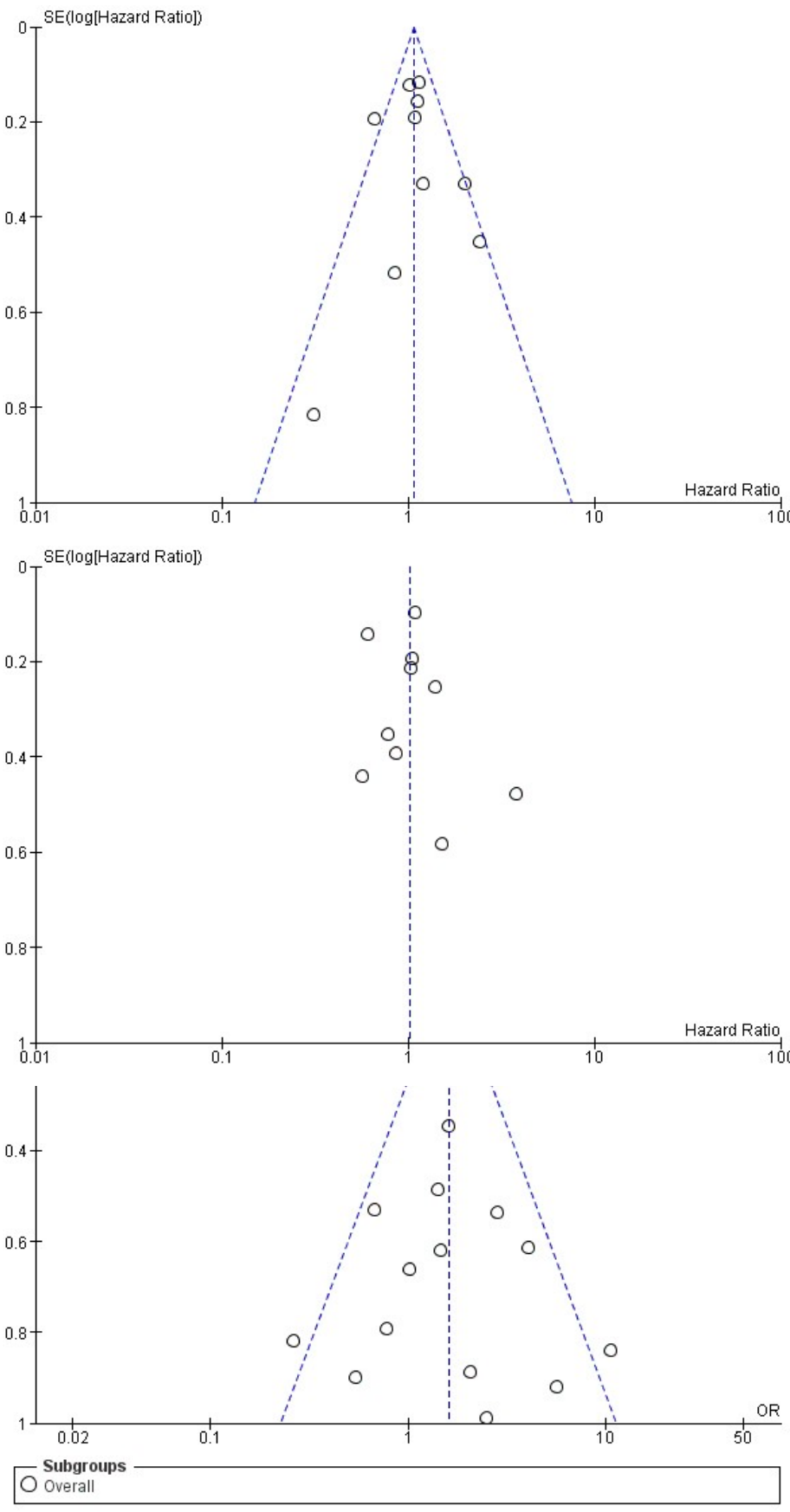

Figure S5. Funnel plots of PFS, OS, and ORR respectively
