# Supplementary material for: Revisiting metformin as a chemotherapy adjuvant: a systematic review and meta-analysis based on recent randomized-controlled trials
Source: Front Oncol. 2026 Sep 9;16:1888506. doi: 10.3389/fonc.2026.1888506 (PMC13639577; doi:10.3389/fonc.2026.1888506)
Supplement: Supplementary file 2 [file Table1.docx]

|  | **D1** | **D2** | **D3** | **D4** | **D5** | **Overall** |
| --- | --- | --- | --- | --- | --- | --- |
| **Skinner 2021** | Low | Low | Low | Low | Low | Low |
| **Martin-Castillo 2018** | Some concerns | Some concerns | High | Low | Some concerns | High |
| **Pimentel 2019** | Low | Low | Low | Low | Low | Low |
| **Kordes 2015** | Low | Low | Low | Low | Low | Low |
| **Marrone 2018** | Low | Low | Low | Low | Low | Low |
| **Yoon 2023** | Low | Low | Low | Low | Low | Low |
| **Essa 2022** | Some concerns | Some concerns | Low | Low | Some concerns | Some concerns |
| **Salah 2021** | Some concerns | Some concerns | Low | Low | Low | Some concerns |
| **Vernieri 2019** | High | Low | Low | Low | Low | High |
| **Reni 2016** | Low | Low | Low | Low | Low | Low |
| **Sayed 2015** | Some concerns | Some concerns | Low | Low | Low | Some concerns |
| **Tsakiridis 2021** | Low | Low | Low | Low | Low | Low |
| **Hegazy 2024** | Low | Low | Low | Low | Low | Low |
| **Nanni 2018** | Low | Low | Low | Low | Low | Low |
| **Zheng 2019** | Some concerns | Some concerns | Low | Low | Some concerns | Some concerns |
| **Lee 2020** | Some concerns | Some concerns | Low | Low | Some concerns | Some concerns |
| **Singh 2016** | High | Some concerns | Low | Low | Some concerns | High |
| **El-Khayat 2021** | Some concerns | Some concerns | Some concerns | Low | Some concerns | Some concerns |
| **Barakat 2022** | Some concerns | Low | Low | Low | Some concerns | Some concerns |
