# Supplementary material for: Revisiting metformin as a chemotherapy adjuvant: a systematic review and meta-analysis based on recent randomized-controlled trials
Source: Front Oncol. 2026 Sep 9;16:1888506. doi: 10.3389/fonc.2026.1888506 (PMC13639577; doi:10.3389/fonc.2026.1888506)
Supplement: Supplementary file 3 [file Table2.docx]

| **Study ID** | **Self-Reported Study Design Label** | **Randomization Method** | **Allocation Concealment** | **Trial Registration / Protocol Identifier** | **Justification for Inclusion as RCT** |
| --- | --- | --- | --- | --- | --- |
| **Barakat et al. (2022)** | Open-label randomized trial | Computer-generated random sequence (1:1) | Sequentially numbered containers | ClinicalTrials.gov (NCT04838184) | Prospective allocation of participants to metformin vs. control arm. |
| **El-Khayat et al. (2021)** | Prospective randomized study | Block randomization | Opaque sealed envelopes | Pan African Clinical Trials Registry | Evaluated comparative intervention arms prospectively via randomized assignment. |
| **Essa et al. (2022)** | Randomized phase II clinical trial | Computer-generated randomization | Central assignment by study coordinator | ClinicalTrials.gov (NCT03901378) | Parallel-group prospective trial with randomized allocation. |
| **Hegazy et al. (2024)** | Open-label randomized trial | Computer-generated random number table | Unclear | ClinicalTrials.gov (NCT05214066) | Prospective parallel assignment comparing treatment vs. control. |
| **Kordes et al. (2015)** | Double-blind, randomized, placebo-controlled trial | Permuted computer-generated block allocation | Hospital pharmacy central allocation | ClinicalTrials.gov (NCT01210911) | Rigorous placebo-controlled double-blind prospective RCT. |
| **Lee et al. (2020)** | Open-label, multi-center randomized phase II trial | Web-based centralized random sequence generation | Central interactive voice/web response system | ClinicalTrials.gov (NCT02080026) | Multi-center prospective randomized study comparing treatment arms. |
| **Marrone et al. (2018)** | Open-label randomized phase II study | Permuted block randomization | Central web-based randomization | ClinicalTrials.gov (NCT01588184) | Prospective parallel randomized study with explicit sequence generation. |
| **Martin-Castillo et al. (2018)** | Open-label, randomized phase II trial (MEET-MIA) | Computerized central randomization (1:1) | Central trial administration system | ClinicalTrials.gov (NCT01611051) | Prospective multi-center randomized comparative trial. |
| **Nanni et al. (2018)** | Randomized phase II study | Computer-generated block sequence | Sealed opaque envelopes | ClinicalTrials.gov (NCT01266486) | Prospective randomized assignment comparing metformin vs. control. |
| **Pimentel et al. (2019)** | Randomized pilot study | Simple computer-generated randomization | Sequentially numbered sealed envelopes | ClinicalTrials.gov (NCT02048384) | Prospective pilot study using true randomized allocation between arms. |
| **Reni et al. (2016)** | Open-label, randomized phase II trial (PACT-17) | Permuted block randomization sequence | Interactive Voice Response System | ClinicalTrials.gov (NCT01167738) | Prospective phase II parallel randomized controlled trial. |
| **Salah et al. (2021)** | Pilot randomized controlled trial | Block randomization (1:1) | Opaque sealed containers | ClinicalTrials.gov (NCT03868215) | Prospective parallel-group trial with true sequence generation. |
| **Sayed et al. (2015)** | Prospective randomized study | Random number table | Unclear | ClinicalTrials.gov / University Registry | Evaluated comparative outcome data following prospective randomized allocation. |
| **Singh et al. (2016)** | Randomized pilot phase II trial | Computer-generated allocation scheme | Central pharmacy dispensation | ClinicalTrials.gov (NCT01510197) | Prospective phase II pilot study with randomized assignment. |
| **Skinner et al. (2021)** | Open-label randomized phase II trial (NRG-LU001) | Permuted block sequence stratified by stage/histology | Central Web-based Registration System (CTS) | ClinicalTrials.gov (NCT02186847) | Multi-center prospective randomized clinical trial. |
| **Tsakiridis et al. (2021)** | Open-label randomized phase II study (OICR-012) | Central block randomization | Central clinical research group allocation | ClinicalTrials.gov (NCT02115464) | Multi-institutional prospective parallel RCT. |
| **Vernieri et al. (2019)** | Open-label, randomized phase II study (MYMET) | Computer-generated 1:1 permuted block sequence | Central trial database | ClinicalTrials.gov (NCT02058384) | Prospective multi-center randomized trial. |
| **Wang et al. (2021)** | Prospective randomized controlled trial | Computerized random number generator | Opaque sealed envelopes | Chinese Clinical Trial Registry (ChiCTR) | Prospective parallel randomized comparative design. |
| **Yoon et al. (2023)** | Multi-center, randomized phase II trial | Web-based centralized random sequence | Central interactive system | ClinicalTrials.gov (NCT03071705) | Multi-center prospective randomized controlled study. |
